# The Iron-Responsive Genome of the Chiton *Acanthopleura granulata*

**DOI:** 10.1093/gbe/evaa263

**Published:** 2020-12-15

**Authors:** Rebecca M Varney, Daniel I Speiser, Carmel McDougall, Bernard M Degnan, Kevin M Kocot

**Affiliations:** 1 Department of Biological Sciences, The University of Alabama, Tuscaloosa, Alabama; 2 Department of Biological Sciences, University of South Carolina, Columbia, South Carolina; 3 Australian Rivers Institute, Griffith University, Nathan, Queensland, Australia; 4 School of Biological Sciences, University of Queensland, Brisbane, Queensland, Australia; 5 Alabama Museum of Natural History, Tuscaloosa, Alabama

**Keywords:** biomineralization, biomaterials, iron, ferritin, iron response element (IRE), mollusc

## Abstract

Molluscs biomineralize structures that vary in composition, form, and function, prompting questions about the genetic mechanisms responsible for their production and the evolution of these mechanisms. Chitons (Mollusca, Polyplacophora) are a promising system for studies of biomineralization because they build a range of calcified structures including shell plates and spine- or scale-like sclerites. Chitons also harden the calcified teeth of their rasp-like radula with a coat of iron (as magnetite). Here we present the genome of the West Indian fuzzy chiton *Acanthopleura granulata*, the first from any aculiferan mollusc. The *A. granulata* genome contains homologs of many genes associated with biomineralization in conchiferan molluscs. We expected chitons to lack genes previously identified from pathways conchiferans use to make biominerals like calcite and nacre because chitons do not use these materials in their shells. Surprisingly, the *A. granulata* genome has homologs of many of these genes, suggesting that the ancestral mollusc may have had a more diverse biomineralization toolkit than expected. The *A. granulata* genome has features that may be specialized for iron biomineralization, including a higher proportion of genes regulated directly by iron than other molluscs. *A. granulata* also produces two isoforms of soma-like ferritin: one is regulated by iron and similar in sequence to the soma-like ferritins of other molluscs, and the other is constitutively translated and is not found in other molluscs. The *A. granulata* genome is a resource for future studies of molluscan evolution and biomineralization.

Significance

Chitons are molluscs that make shell plates, spine- or scale-like sclerites, and iron-coated teeth. Currently, all molluscs with sequenced genomes lie within one major clade (Conchifera). Sequencing the genome of a representative from the other major clade (Aculifera) helps us learn about the origin and evolution of molluscan traits. The genome of the West Indian Fuzzy Chiton, *Acanthopleura granulata*, reveals chitons have homologs of many genes other molluscs use to make shells, suggesting all molluscs share some shell-making pathways. The genome of *A. granulata* has more genes that may be regulated directly by iron than other molluscs, and chitons produce a unique isoform of a major iron-transport protein (ferritin), suggesting that chitons have genomic specializations that contribute to their production of iron-coated teeth.

## Introduction

Animals construct hardened structures by combining organic and inorganic components, a process termed biomineralization. To do so, they secrete proteins that initiate and guide the crystallization of inorganic molecules. Animals also incorporate proteins into biomineralized structures, enhancing their strength and flexibility (Cölfen 2010). Molluscs have long been models for studying the genetic mechanisms associated with biomineralization because they craft a wide range of materials into shells, spines, scales, and teeth (McDougall and Degnan 2018). The ability of molluscs to produce diverse biomineralized structures likely contributes to their remarkable morphological and ecological diversity.

**Fig. 1:**
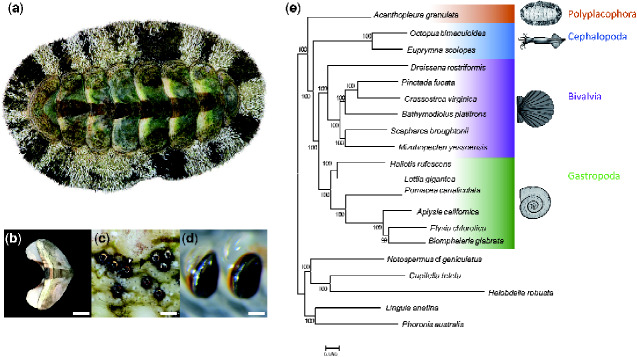
(*a*) The West Indian Fuzzy Chiton *Acanthopleura granulata*. Photograph by David Liittschwager. (*b*) A single shell plate from *A. granulata*. Scale bar indicates 5 mm. (*c*) The eyes (white arrowhead) and aesthete pores (black arrowhead) of *A. granulata*. Scale bar indicates 200 μM. Photograph by David Liittschwager. (*d*) Teeth from the anterior-most region of the radula of *A. granulata*. The larger teeth, used for feeding, are mineralized with iron oxide (orange) and capped with magnetite (black). Scale bar indicates 300 μM. (*e*) A genome-based phylogeny of Mollusca showing chitons as sister to all other molluscs with available genomes.

Chitons (Polyplacophora, fig. 1*A*) are a promising model for investigating mechanisms of biomineralization because they build diverse mineralized structures distinct from those of other molluscs (supplementary fig. 1, Supplementary Material online). The shells of all molluscs are composed of calcium carbonate (CaCO_3_), commonly in its crystal forms aragonite or calcite. Most molluscs build shells with alternating layers of aragonite and calcite, and many add an innermost layer of brick-like aragonite discs known as nacre. In contrast, chitons construct eight interlocking shell plates (fig. 1*B*) exclusively from aragonite and do not produce nacre. Also unlike other molluscs, chitons embed a network of sensory structures, termed aesthetes, into their shell plates. In some species, the aesthete network includes eyes with image-forming lenses made of aragonite (Speiser et al. 2011; Li et al. 2015) (fig. 1*C*). To protect the soft girdle tissue surrounding their shell plates, chitons produce scale- or spine-like sclerites, which are also made of aragonite (Schwabe 2010; Sigwart et al. 2014; Checa et al. 2017).

Chitons biomineralize their teeth (fig. 1*D*) from a unique combination of materials. Most molluscs have a feeding organ, the radula, that bears rows of teeth built from chitin and, in many species, hardened with minerals such as calcium carbonate or silica. Chitons instead harden the cores of their teeth with calcium phosphate (as apatite), and then reinforce their cutting edges with iron (as magnetite) (Lowenstam 1962). These iron coatings allow chitons to scrape algae from rocks without rapidly dulling or damaging their teeth. Chitons produce new teeth throughout their lives, making new rows within days (Shaw et al. 2002; Joester and Brooker 2016). To make new teeth, chitons continuously sequester iron from their diet and circulate high concentrations of iron in their hemolymph (Kim et al. 1989; Shaw et al. 2002, 2010). Continuous iron biomineralization presents a physiological challenge to chitons because free iron causes oxidative stress (Dixon and Stockwell 2014).

To date, most investigations of biomineralization in molluscs have focused on species from the classes Bivalvia and Gastropoda. These, together with Monoplacophora, Cephalopoda, and Scaphopoda make up the clade Conchifera. The sister clade to Conchifera is Aculifera, which is made up of Polyplacophora, Solenogastres, and Caudofoveata (the latter two classes referred to collectively as Aplacophora). Conchifera and Aculifera diverged approximately 550 Ma (Vinther et al. 2012; Kocot et al. 2020). To make robust predictions about molluscan evolution, reconstructions of ancestral character states must include information from both conchiferans and aculiferans (Sigwart and Sutton 2007; Kocot et al. 2011; Smith et al. 2011; Vinther et al. 2012). Despite increasing numbers of sequenced molluscan genomes (e.g., Takeuchi et al. 2012; Zhang et al. 2012; Simakov et al. 2013; Albertin et al. 2015; Gómez-Chiarri et al. 2015; Modica et al. 2015; Kenny et al. 2015; Barghi et al. 2016; Davison et al. 2016; Murgarella et al. 2016; Adema et al. 2017; Du et al. 2017; Nam et al. 2017; Schell et al. 2017; Sun et al. 2017; Wang et al. 2017; Calcino et al. 2018; Gerdol et al. 2018; Li et al. 2018; Liu et al. 2018; Renaut et al. 2018; Belcaid et al. 2019; Cai et al. 2019; Kijas et al. 2019; Masonbrink et al. 2019; McCartney et al. 2019; Zarrella et al. 2019; Sun et al. 2020), genomic resources for aculiferans remain unavailable. To advance the study of molluscan evolution and to better understand the genetic mechanisms of biomineralization, we sequenced the genome of the West Indian fuzzy chiton *Acanthopleura granulata*. Exploring the *A. granulata* genome allowed us to: 1) identify genes chitons may use to build their shell plates, sclerites, and teeth; 2) seek genomic signatures associated with the biomineralization of iron and the mitigation of iron-induced oxidative stress; and 3) better understand the origin and evolution of biomineralization in molluscs.

## Results and Discussion

We sequenced the genome of a single male specimen of *A. granulata*. We combined reads from one lane of Illumina HiSeq X paired-end sequencing (124 Gb of 2 × 150 bp reads, ∼204 × coverage) with reads from four Oxford Nanopore flowcells run on the GridION platform (22.87 Gb, 37× coverage). Using the hybrid assembler MaSuRCA and optical mapping, we produced a haploid genome assembly for *A. granulata* that is 606.9 Mp, slightly smaller than the 743 Mp haploid genome size estimated by flow cytometry (Roebuck 2017). The assembled *A. granulata* genome consists of 87 scaffolds ranging in size from 50.9 to 0.05 Mb, plus a single mitochondrial genome of 15,665 bp. Previous studies across chitons found haploid chromosome numbers of anywhere from 6 to 16, and noted the presence of micro-chromosomes (Odierna 2008). Several of the scaffolds from the *A. granulata* genome are similar in length to intact chromosomes from other molluscs (Sun et al. 2020; Bai et al. 2019), so we are confident that, at a minimum, several scaffolds represent complete arms of chromosomes. To verify completeness of the assembly, we mapped genomic short-read data to the genome; 85.31% of reads mapped perfectly, so we are confident the assembly encompasses a majority of sequencing data. The *A. granulata* genome has an N50 value of 23.9 Mp and a BUSCO completeness score of 97.4%, making it more contiguous and complete than most currently available molluscan genomes (supplementary fig. 2; supplementary table 1; visualized in supplementary fig. 3, Supplementary Material online).

We generated gene models for *A. granulata* by 1) sequencing transcriptomes from eight different tissues from the same specimen used for genome sequencing, 2) combining these transcriptomes into a single assembly and aligning the combined transcriptome to the genome, and 3) training de novo gene predictors using both our combined transcriptome and protein sequences predicted from the transcriptomes of other chitons. Following these steps, we produced a set of 81,691 gene models that is 96.9% complete according to a BUSCO transcriptomic analysis. This score is similar to the completeness score of the *A. granulata* genome, so it is likely this set of gene models missed few genes, if any, in the genome assembly. However, of the BUSCO genes expected to be single copy in all animals, 17.2% were represented by multiple gene models. Using Markov clustering to eliminate redundant isoforms, we generated a reduced set of 20,470 gene models that is 94.7% complete. In this smaller set of gene models, only 0.5% of the BUSCO genes have multiple copies, supporting Markov clustering as an effective method for reducing the redundancy of gene models. To characterize proteins based on shared functional domains and sequence similarity, we analyzed the set of 20,470 gene models with InterProScan. We identified at least one GO term for 12,301 genes and a Pfam match for 15,710 genes (4,792 unique PFAM domains). We also conducted a KEGG analysis and identified 7,341 proteins that may be homologous to those that are parts of characterized molecular pathways.

To provide a robust data set for phylogenetic analysis and gene family evolution analyses, we identified homologous genes shared between *A. granulata* and other molluscs. We used the larger set of gene models from *A. granulata* to ensure a more complete input data set, knowing that any duplicate gene models for the same locus would cluster within the same orthologous group. We compared gene models from the *A. granulata* genome to those from the genomes of 19 other lophotrochozoans, including 14 molluscs, 2 annelids, 1 brachiopod, 1 phoronid, and 1 nemertean. This resulted in 59,276 groups of homologous sequences including 3,379 found in all 20 genomes.

We used a tree-based approach to identify orthologous genes shared among all 20 taxa and reconstructed molluscan phylogeny using the 2,593 orthologs present in at least 17 of the 20 genomes we searched. This data set totaled 950,322 amino acid positions with 16.2% missing data. We recovered *A. granulata* as the sister taxon of all other molluscs with sequenced genomes (fig. 1*E*). We conducted an additional phylogenetic analysis that included more taxa by using transcriptomes in addition to genomes and recovered *Acanthopleura* within the family Chitonidae in the order Chitonida, consistent with recent phylogenetic studies of chitons based on fewer loci (Irisarri et al. 2020); (supplementary fig. 4, Supplementary Material online).

### The *A. granulata* Genome Differs from Conchiferan Genomes in Content and Organization

The *A. granulata* genome has a heterozygosity of 0.653%, making it one of the least heterozygous molluscan genomes sequenced to date (supplementary fig. 5, Supplementary Material online). High heterozygosity in animals is often attributed to high rates of gene flow associated with broadcast spawning and far-dispersing larvae (Solé-Cava and Thorpe 1991), and it is frequently noted as an obstacle to genome assembly in molluscs (Zhang et al. 2012; Wang et al. 2017; Powell et al. 2018; Thai et al. 2019). We expected the genome of *A. granulata* to have high heterozygosity because this species of chition is a broadcast spawner with a wide geographic range (Glynn 1970). To compare heterozygosity across molluscs, we selected a set of high-quality molluscan genomes for which short-read data are available (supplementary table 1, Supplementary Material online). Using k-mer-based analysis, we found the highest heterozygosity among the seven genomes we analyzed was 3.15% in the blood clam *Scapharca broughtonii*, and the other genomes had heterozygosities between those of *A. granulata* and *S. broughtonii*. Our findings indicate that heterozygosity may be influenced by more than an animal’s reproductive mode, larval type, and geographic range (Romiguier et al. 2014; supplementary table 2, Supplementary Material online), and that molluscan genomes should not be assumed to have high heterozygosity.

The *A. granulata* genome is arranged differently than other molluscan genomes and has fewer repetitive elements. Compared with a non-molluscan lophotrochozoan, *Lingula anatina* (a brachiopod), *A. granulata* has more repetitive elements of certain types in its genome. Conversely, *A. granulata* has fewer of many types of repetitive elements in its genome than conchiferan molluscs (supplementary table 3 and supplementary fig. 6, Supplementary Material online). This suggests multiple proliferations of repetitive elements during molluscan evolution. Repetitive elements contribute to structural changes in genomes by providing breakpoints that increase the likelihood of chromosomal rearrangements (Weckselblatt and Rudd 2015). Consistent with this prediction, synteny is lower between *A. granulata* and all conchiferan molluscs we examined than it is between any two of these conchiferans, and the genomes of conchiferans and *A. granulata* have little synteny with the genome of *L. anatina* (supplementary fig. 7, Supplementary Material online). A recent study of bilaterian ancestral linkage groups (ALGs) found greater synteny in ALGs between a scallop and non-molluscs than between other bivalves and the same non-molluscs (Wang et al. 2017), but not all molluscan classes were examined for ALGs. Molluscan genomes appear to rearrange frequently across evolutionary time, and perhaps rearrange more frequently in conchiferans due to the proliferation of repetitive elements.

**Fig. 2:**
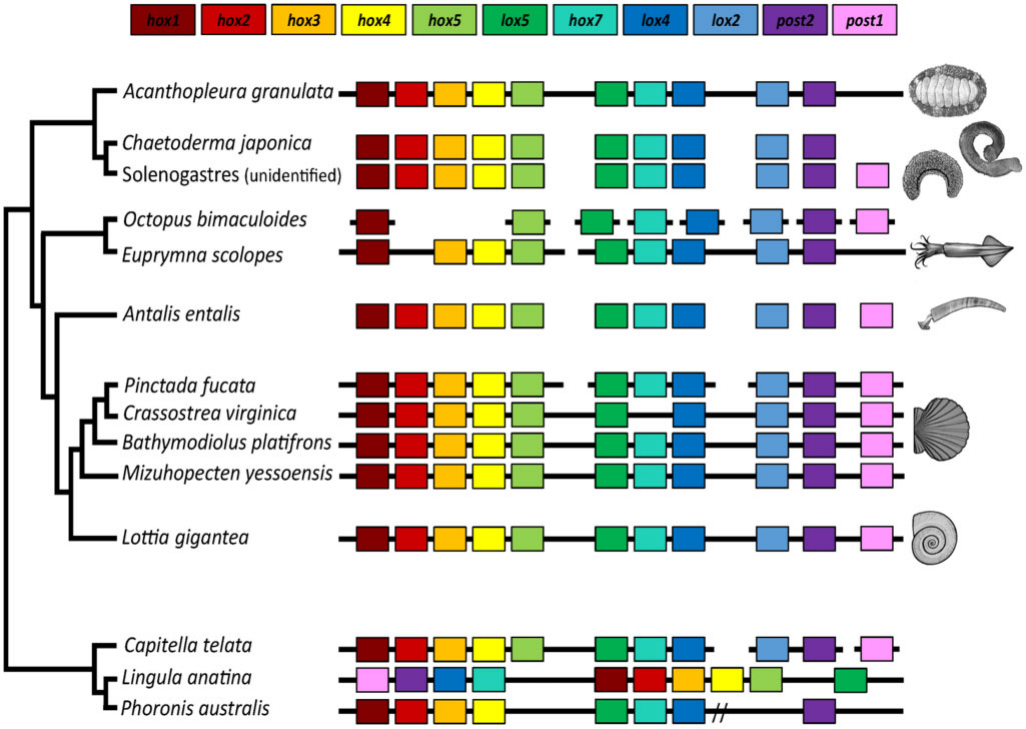
Synteny of Hox genes between *Acanthopelura granulata* and other taxa. The presence of a gene is indicated by a box of the corresponding color. Continuous black lines indicate that the Hox genes in this species were located on a contiguous genomic scaffold. Broken black lines indicate that gene(s) are located on multiple genomic scaffolds. A double slash indicates genes are located on a single contiguous scaffold but separated by greater distances than those in most other taxa.

The Hox cluster is a widely conserved set of regulatory genes that together contribute to the patterning of the anterior-posterior axes in bilaterian animals. In lophotrochozoans, the genes are typically collinear, beginning with *Hox1* and ending with *Post1*. Although several gastropods and bivalves possess intact Hox clusters, this cluster is dispersed in some bivalves and some cephalopods (Albertin et al. 2015; Barucca et al. 2016; Belcaid et al. 2019; Wang et al. 2017; Peng et al. 2020; Gąsiorowski and Hejnol 2020). We found the Hox cluster of *A. granulata* is collinear with the Hox clusters of most other molluscs but lacks *Post1* (fig. 2). Living chitons are divided into orders Lepidopleurida and Chitonida, and Chitonida is divided into suborders Acanthochitonina and Chitonina, with *A. granulata* belonging to the latter (Sirenko 2006). A previous study found two species of chitons from suborder Acanthochitona are missing the Hox gene *Post1* (Wanniger and Wollesen 2019; Huan et al. 2019). *Post1* is present in aplacophorans and in almost all conchiferan molluscs (Iijima et al. 2006). This suggests *Post1* was lost in either the common ancestor of Chitonida or the common ancestor of all living chitons. In conchiferan molluscs, *Post1* helps specify the posterior of an animal during development and helps pattern shell formation (Lee et al. 2003; Fröbius et al. 2008; Schiemann et al. 2017; Huan et al. 2019). In the absence of *Post1*, *A. granulata* and other chitons must use other regulatory genes to help pattern their body axes and biomineralized structures.

### *A. granulata* Shares Many Biomineralization Genes with Conchiferan Molluscs

We expected chitons to lack many genes previously identified from biomineralization pathways in conchiferans because their shell plates and sclerites lack both calcite and nacre (materials that most conchiferans incorporate into shells). We determined orthogroups across *A. granulata* and several other molluscs with whole-genome data, and were surprised to find orthologs in the *A. granulata* genome of many genes that contribute to biomineralization in conchiferans (supplementary table 4 and supplementary fig. 8, Supplementary Material online; supplementary data available via Dryad). For example, we found two homologs of *Pif* in the *A. granulata* genome. In pterid bivalves, *Pif* mRNA encodes a peptide that is cleaved into two functional proteins, PIF97 and PIF80 (Suzuki et al. 2009). These proteins have different roles in biomineralization: PIF80 binds nacre and aids in nacre formation (Suzuki et al. 2009), whereas PIF97 binds to chitin and guides the growth of calcium carbonate crystals (Suzuki et al. 2013). One homolog of *Pif* in the *A. granulata* genome, g24122, codes for a protein that is similar to PIF97 but not to PIF80, suggesting that the *A. granulata Pif* does not code for a protein similar to PIF80. On a phylogeny including protein sequences from orthogroups with sequences similar to PIF, as well as known PIF protein sequences identified in other molluscs, *A. granulata* PIF falls in a monophyletic clade with other known PIF proteins to the exclusion of proteins with similar sequences that are not PIF (supplementary fig. 8*A*, Supplementary Material online, clade labeled PIF). The second homolog of *Pif* in the *A. granulata* genome, g24110, codes for a protein that is “PIF-like.” A “PIF-like” protein in *Lymnaea stagnalis* is associated with shell biomineralization (Ishikawa et al. 2020). On a phylogeny including PIF and “PIF-like” sequences, the *A. granulata* “PIF-like” homolog falls in a clade with the *L. stagnalis* “PIF-like” protein sequence, and not in the clade with PIF. This suggests that “PIF-like” proteins are the products of genes that are not *Pif* (supplementary fig. 8*A*, Supplementary Material online, clade-labeled PIF-like). Further supporting the distinction between *Pif* and *Pif-like* genes, we found that the *Pif* and “*PIF-like*” genes are located on different scaffolds in the genome of *A. granulata*, suggesting the genes have independent origins.

We examined the expression of transcripts for the *A. granulata Pif* and *Pif-like* homologs and found both homologs have similar patterns of mRNA expression*.* The expression of the mRNA from the *Pif* (*Pif97*) homolog was highest in girdle tissue and lowest in the radula, suggesting that the protein produced by *Pif* may play a role in sclerite formation in *A. granulata* and perhaps in other chitons (supplementary table 5, Supplementary Material online). The expression of mRNA from the “*Pif-like*” homolog was also highest in the girdle of *A. granulata* compared with other tissues, so the “PIF-like” protein encoded by these transcripts may also be involved in the production of sclerites. We hypothesize that the last common ancestor of extant molluscs used a protein similar to PIF97 to help build mineralized structures, and that production of PIF80 is novel to bivalves. Further study is needed to determine the independent evolution and functions of PIF and “PIF-like” proteins in molluscan biomineralization.

The ancestral mollusc likely produced mineralized structures, but whether the ancestral mollusc had a single shell, multiple shell plates, or sclerites remains a matter of debate (Kocot 2013; Scherholz et al. 2013; Vinther et al. 2017; Giribet and Edgecombe 2020). Molluscs form mineralized structures by making extracellular matrices from organic components, such as polysaccharides and proteins, and then hardening them with minerals (Furuhashi et al. 2009). Similarities between the extracellular matrices of different biomineralized structures suggest these structures share developmental mechanisms. The *A. granulata* genome includes genes that code for proteins characterized from the extracellular matrices of conchiferans. Chitin is a major component of the extracellular matrices of all molluscan shells and radulae, and the *A. granulata* genome contains genes for chitin synthase, chitinase, and chitin-binding proteins. Additionally, we found homologs of genes that code for dermatopontin (supplementary fig. 8*B*, Supplementary Material online) and laminin (supplementary fig. 8*C*, Supplementary Material online), two proteins expressed in the extracellular matrices of conchiferans that increase the elasticity and flexibility of their shells (Gaume et al. 2014; supplementary table 4 and supplementary fig. 8, Supplementary Material online). In phylogenetic analyses, the *A. granulata* homologues identified here group with protein sequences from known shell matrix components in other taxa, indicating that these proteins may also be involved in biomineralization in chitons.

Silk-like structural proteins are components of many biological materials, including shells (Eisoldt et al. 2011; McDougall et al. 2016; Xu et al. 2016), and several *A. granulata* genes are similar to genes known to code for silk-like proteins. These proteins are “silk-like” because they contain highly repetitive sequences of amino acids that fold into secondary structures (commonly β-pleated sheets) that impart flexibility, a phenomenon first documented in spider silk (Lewis 2006; Eisoldt et al. 2011). Silk-like domains can facilitate the precipitation and crystallization of minerals that help form structures such as bones and shells (Xu et al. 2016). We found 31 genes in the *A. granulata* genome that code for proteins with silk-like domains, 23 of which have high sequence similarity to genes associated with biomineralization in other molluscs (supplementary table 6, Supplementary Material online). We found 27 of these 31 genes from the *A. granulata* genome code for proteins with signal peptides, indicating they may be secreted as part of the extracellular matrix during biomineralization (supplementary table 6, Supplementary Material online). We also found genes that code for three collagens, one chitinase, and one carbonic anhydrase, all possible contributors to shell formation and repair in chitons (Patel 2004; supplementary table 6, Supplementary Material online). Several of the genes encoding proteins with silk-like domains are highly expressed in the girdle of *A. granulata*, suggesting a role in the mineralization of sclerites (supplementary fig. 9, Supplementary Material online).

### *A. granulata* Has More Genes with Iron Response Elements than Other Molluscs

**Fig. 3:**
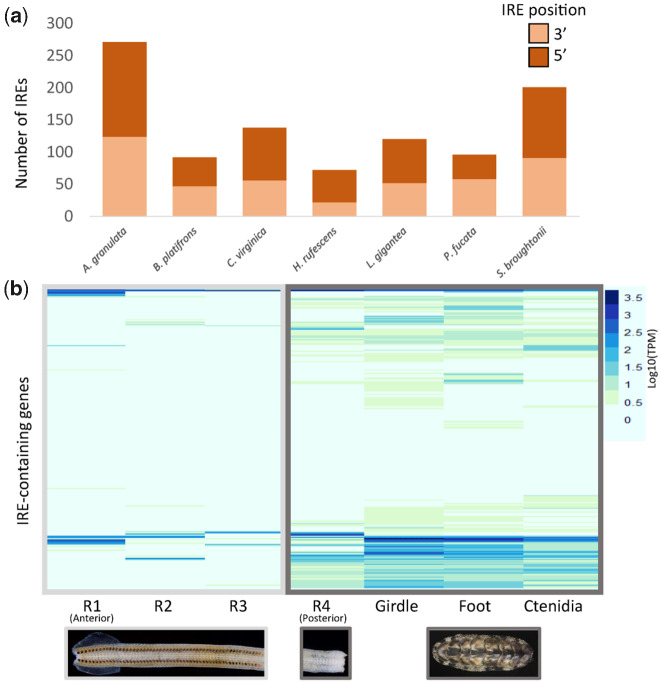
IREs in the *Acanthopleura granulata* genome. (*a*) The number of IREs in several molluscan gene model sets, and relative proportions of 5′ and 3′ IREs. *A. granulata* has more IREs than any other molluscs examined, but the relative proportions of 5′ and 3′ IREs appear consistent across molluscan genomes. (*b*) The relative expression (log10(TPM)) of transcripts containing IREs in the different tissues of *A. granulata*. The radula is divided into four developmentally distinct regions: R1, the most anterior region, contains teeth used for feeding; R2 contains teeth that are developed but are not yet used for feeding; R3 contains developing teeth that contain iron oxide; and R4, the most posterior region, contains developing teeth that have yet to be coated with iron. We found lower expression of most IRE-containing genes in the anterior regions of the radula.

Chitons have more iron in their circulatory fluid (hemolymph) than any other animal studied to date (Kim et al. 1988). Iron presents physiological challenges to animals because it can cause oxidative stress. We hypothesize that the ability of chitons to biomineralize iron requires them to respond quickly to changes in concentration of this potentially toxic metal. To assess the iron-responsiveness of the *A. granulata* genome, we searched it for iron response elements (IREs), three-dimensional hairpin structures that form in either the 3′ or 5′ untranslated regions (UTRs) of mRNA molecules and control translation via the binding of iron regulatory protein (IRP; supplementary fig. 10, Supplementary Material online). We also examined IREs in several high-quality molluscan genomes that include UTRs as part of their available annotation data. All of the molluscan genomes we examined had similar proportions of 3’ to 5’ IREs (fig. 3*A*). Despite having the fewest gene models, the genome of *A. granulata* has more IREs than the genomes of any other mollusc we examined. We predicted 271 IREs in the *A. granulata* genome, compared with an average of 119 IREs across other molluscan genomes (supplementary table 7, Supplementary Material online). The highest number of predicted IREs in a conchiferan came from the genome of the blood clam *S. broughtonii*, which had 201. The blood clam is so named because it is one of relatively few molluscs that produces hemoglobin for use as a respiratory pigment (Kawamoto 1928; Manwell 1963; Read 1966; Collett and O’Gower 1972; Bai et al. 2019). We expect *A. granulata* and *S. broughtonii* have more IREs in their genomes than other molluscs because they must absorb and transport larger amounts of iron to produce iron-coated teeth and hemoglobin, respectively.

We next examined the expression of genes that contain a 5′ IRE across tissues of *A. granulata*, genes that would be expected to be expressed at higher levels in the presence of iron than in the absence of iron. We divided the radula of *A. granulata* into four regions based on the amount of iron present in each (supplementary fig. 1*C*, Supplementary Material online). We found a number of genes with 5′ IREs that are expressed at relatively high levels in the three iron-rich anterior regions of the radula compared with other tissues of *A. granulata* (fig. 3*B*). We then asked if these genes might have roles in the biomineralization of the radula. We used Gene Ontology (GO) analysis to compare the functions predicted for the protein sequences coded by the genes with 5′ IREs to the functions predicted for the protein sequences coded by the full set of genes from the *A. granulata* genome. We found that genes with a 5′ IRE that are highly expressed in the anterior of the radula are more likely than other genes to be associated with the molecular functions “response to inorganic substance,” “response to calcium ion,” and “response to metal ion” (supplementary fig. 12, Supplementary Material online). This suggests that genes with a 5′ IRE that are highly expressed in the radula may be involved in the biomineralization of the apatite (calcium phosphate) cores of teeth and their magnetite (iron) caps. A previous study by Nemoto et al. identified a novel biomineralization protein (RTMP1) in the radula of another species of chiton (*Cryptochiton stelleri*), and proposed that RTMP1 played a role in iron biomineralization (Nemoto et al. 2019). We examined the mRNA of RTMP1 in *C. stelleri* and did not detect an IRE in either its 5′ or 3′ UTR. Thus, there are genes that may contribute to iron biomineralization in the chiton radula whose expression levels are not influenced by IREs.

### Two Isoforms of Ferritin May Provide Chitons with Tissue-Specific Protection from Oxidative Stress

**Fig. 4:**
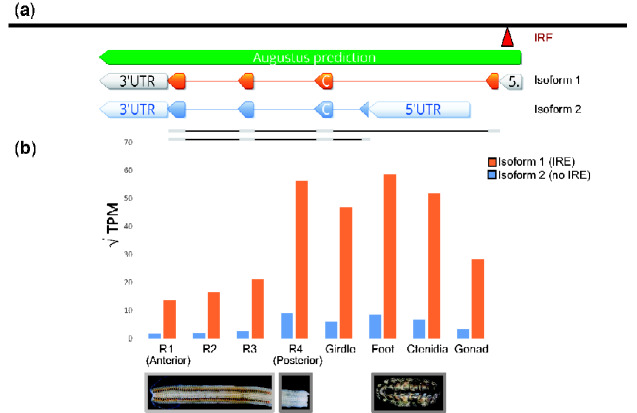
The two isoforms of heavy-chain ferritin present in *Acanthopleura granulata*. (*a*) The locations of the transcription initiation sites and exons of isoform 1 of ferritin (orange, above) and isoform 2 of ferritin (blue, below). A 5′ IRE (red) is present in the 5′ UTR of isoform 1, but not in isoform 2. (*b*) Relative expression of both isoforms of ferritin across *A. granulata* tissues. The radula is divided into four developmentally distinct regions as in fig. 3. Isoform 1 is expressed more highly throughout the body than isoform 2. Isoform 2 is expressed at lower levels in the anterior (iron-rich) regions of the radula than in other tissues. We visualized data with (√(TPM)) to allow both data ranges to appear legibly on the same graph.

All metazoans require iron. However, free iron poses a threat to animals because it catalyzes the production of reactive oxygen species that inflict damage on DNA and tissues (Dixon and Stockwell 2014). To transport iron safely, metazoans use the iron-binding protein ferritin. Previous work suggests that chitons use ferritin to transport iron to their radula (Kim et al. 1988). An IRE is present in the 5′ UTR of the heavy chain (or soma-like) ferritin mRNA that is expressed by all metazoans (Piccinelli and Samuelsson 2007). We found two isoforms of heavy chain ferritin in our gene models for *A. granulata*: a first isoform (isoform 1) that contains the conserved 5′ IRE, and a second isoform (isoform 2) that does not (fig. 4*A*;supplementary table 8, Supplementary Material online).

Isoform 1 of ferritin from *A. granulata* contains an IRE in its 5′ UTR, allowing this isoform to be translated only in the presence of free iron. By regulating the translation of ferritin, cells can transcribe ferritin mRNA continuously so they are primed to produce large quantities of ferritin protein rapidly if conditions require it. If no free iron is present, IRP will bind to the IRE and block translation. We found isoform 1 of ferritin is expressed at high levels in all the non-radula transcriptomes we sequenced for *A. granulata*, including those for the foot, girdle, gonad, and ctenidia. Isoform 1 is also expressed in all four regions of the radula, although at lower levels in the anterior, iron-rich regions than in other tissues (fig. 4*B*). Thus, when *A. granulata* needs to bind excess iron, it may be able to rapidly produce isoform 1 of ferritin protein throughout its body. We examined other mollusc genomes and transcriptomes and found a ferritin isoform present in all of them that is similar to *A. granulata* isoform 1 and contains the 5′ IRE (supplementary table 8, Supplementary Material online).

Isoform 2 of ferritin in *A. granulata* lacks the 5′ IRE present in isoform 1. We identified an alternative transcription initiation site downstream of ferritin exon 1 in the *A. granulata* genome. Isoform 2 of ferritin, initiated at this downstream site, contains a different exon 1 than isoform 1 of ferritin, but shares exons 2–4 with isoform 1 (fig. 4). We examined other molluscan genomes and transcriptomes and did not find evidence for expression of a ferritin isoform similar to *A. granulata* isoform 2 (data available on Dryad). In *A. granulata*, isoform 2 is expressed at lower levels than isoform 1 throughout all body tissues (foot, girdle, gonad, ctenidia). Isoform 2 is expressed in the posterior region of the radula where iron mineralization does not occur, but its expression is almost undetectable in the iron-rich regions of the radula. Without the 5′ IRE, translation of the mRNA of isoform 2 is not blocked in the absence of free iron. We hypothesize that chitons use isoform 2 of ferritin to produce a low level of ferritin protein constitutively in tissues outside their radula as protection from the high concentrations of iron in their circulatory fluid. The 5′ IRE in ferritin is an important regulatory mechanism for protein production. In rats, for example, the expression of ferritin mRNAs is relatively constant across tissues but protein levels vary (Rogers and Munro 1987). Further, mutations in the 5′ IRE of ferritin cause hyperferritinemia in mammals, an iron-related medical condition caused by an overproduction of ferritin protein (Thomson et al. 1999).

## Conclusions

The *A. granulata* genome is the first available genome for any chiton or any aculiferan. The information it provides improves our understanding of the evolution of biomineralization across Mollusca as well as lineage-specific innovations within chitons. Chitons are a valuable system for investigating biomineralization because they produce shell plates, sclerites, and iron-clad teeth. The unique combination of structures produced by chitons makes the *A. granulata* genome a resource for future studies of biomineralization. Although many genes involved in molluscan shell secretion are rapidly evolving (Jackson et al. 2006; Kocot et al. 2016), we were able to identify homologs of many conchiferan biomineralization genes in the *A. granulata* genome. The expression of several genes associated with conchiferan shell secretion in the girdle of *A. granulata* suggests these genes may function in sclerite biomineralization in chitons. This suggests a common underlying molecular mechanism for the biomineralization of conchiferan shells and aculiferan sclerites, structures known to share some developmental pathways even though they arise via different cell lineages (Wollesen et al. 2017).

All metazoans require iron, but they must balance iron use against potential oxidative damage. Regulating iron is a particular concern for chitons because they biomineralize their teeth with magnetite. The genome of *A. granulata* contains more genes with IREs than the genome of any other mollusc examined to date, indicating it has a larger proportion of genes regulated directly by iron. We identified two isoforms of ferritin in *A. granulata*, one that is iron-responsive and a second that is constitutively translated. We propose the second isoform of ferritin protects tissues outside the radula from oxidative stress by binding free iron. Chitons are an emerging model for studies of both biomineralization and iron homeostasis. The *A. granulata* genome will aid future studies by suggesting specific proteins and pathways to target with comparative studies of gene expression and gene manipulation.

## Materials and Methods

### Specimen Collection and Preservation

We collected a single male specimen of *Acanthopleura granulata* from Harry Harris State Park in the Florida Keys (Special Activity License #SAL-17-1983-SR). We cut the majority of the foot into ∼1-mm^2^ cubes and froze them at −80 °C. We froze additional pieces of foot, girdle (dissected such that the tissue sample would not contain shell secretory tissue), ctenidia, gonad, and radula in RNAlater and stored them at −80 °C as well.

### Genome and Transcriptome Sequencing

We extracted high molecular weight DNA from frozen samples of foot tissue from *A. granulata* using a CTAB-phenol chloroform method (available on Dryad). We cleaned DNA for short-read generation with the Zymo Clean and Concentrator Kit. For library preparation and sequencing, we sent cleaned DNA to the Genomics Services Lab at HudsonAlpha (Huntsville, AL), where it was sheared with a Covaris M220 to an average fragment size of 350 bp. These fragments were used to prepare an Illumina TruSeq DNA PCR-Free library, which was sequenced using one lane of an Illumina HiSeq X (2 × 150 bp paired-end reads).

For long-read sequencing, we cleaned DNA and enriched it for higher-molecular weight fragments by performing two sequential purifications using 0.4× AmPureXP magnetic beads. We generated long reads with four flow cells on an Oxford Nanopore Technologies GridION. We prepared two sequencing libraries with ligation kit LSK-108 and sequenced them on FloMin106 (R9.4.1) flow cells. We prepared the other two sequencing libraries with the updated ligation kit LSK-109 and sequenced them on R9.4.1RevD flow cells. We generated 2.19, 4.41, 7.87, and 8.4 Gb, respectively, across the four flow cells, for a total of 22.87 Gb, or >20× coverage with long-reads. Reads were base called with Guppy 4.0. We trimmed long reads with PoreChop (Wick 2018), which was set to remove chimeras (approximately 0.0005% of reads) and all residual adapter sequences.

To generate transcriptomes, we used the Omega Bio-tek EZNA Mollusc RNA Kit to extract RNA from girdle, ctenidia, gonad, foot, and four regions of radula (representative of visibly different stages of iron mineralization) of the same individual of *A. granulata* we used for genome sequencing. We synthesized and amplified complementary DNA (cDNA) from each tissue using the SmartSeq v4 Ultra Low-input RNA kit (Clontech) from 1 ng of input RNA with 17 cycles of PCR. We created eight dual-indexed sequencing libraries with the Illumina Nextera XT kit, using 1 ng of input cDNA. We sent the eight libraries to Macrogen (Seoul, South Korea) where they were pooled and sequenced on one lane of an Illumina HiSeq 4000 (2 × 100 bp paired-end reads). Using a similar approach, we generated transcriptomes from several additional species of chitons and from a variety of tissues: *Chiton marmoratus* (decalcified valve), *Chiton tuberculatus* (radula), *Acanthopleura gemmata* (girdle and mantle), *Cryptoplax larvaeformis* (girdle), *Callochiton* sp. (whole animal), *Chaetopleura apiculata* (girdle), *Hanleya hanleyi* (mantle), *Leptochiton asellus* (mantle) *Nutallochiton mirandus* (girdle), *Tonicia schrammi* (decalcified valve), *Katharina tunicata* (radula), and *Lepidozona mertensi* (radula).

### Genome and Transcriptome Assembly and Quality Assessment

We initially assembled the chiton genome with MaSuRCA v. 3.3.5 (Zimin et al. 2013), which consolidates paired-end data into super reads and then uses long-read data to scaffold and gap-fill. This produced an assembly with 2,858 contigs. We filtered and collapsed heterozygous contigs with Redundans v. 0.14a (Pryszcz and Gabaldón 2016), decreasing the assembly to 1,285 contigs. To ensure that no contigs were incorrectly removed, we verified that all pre-Redundans contigs mapped to the post-Redundans assembly with bowtie2 (Langmead and Salzberg 2012); all contigs mapped and thus nonredundant data were not deleted. To help decontaminate reads and contigs, we used the Blobtools2 Interface to create blob plots (Blaxter and Challis 2018). Because Blobtools uses the NCBI nucleotide database to determine the identity of each scaffold, and chordate sequences vastly outnumber molluscan sequences in NCBI, Blobtools identified a large proportion of scaffolds as chordate. We identified contaminants as sequences that differed from the majority of scaffolds in both GC content and coverage and used BLAST to verify these sequences as bacterial before removing them from the assembly.

We scaffolded this reduced assembly with one lane of Bionano SAPHYR optical mapping, using two enzymes (BssSI and DLE1) and Bionano Solve v3.4’s scaffolding software, which resulted in 87 scaffolds. We ran REAPR v. 1.0.18 (Hunt et al. 2013), which map short read data and collect mapping statistics simultaneously, to determine accuracy of the assembly overall relative to all short-read data generated, and found despite reducing heterozygosity in the final assembly, 85.31% of paired-end reads map perfectly back to the genome assembly, indicating a complete genome assembly relative to the paired-end data.

To assess our genome assembly, we ran QUAST v. 5.0.2 (Gurevich et al. 2013). We assessed genome completeness with BUSCO v. 4.0.2 (Simão et al. 2015), using the proportions of nuclear protein-coding genes thought to be single-copy in the genomes of diverse metazoans (Metazoa odb9 data set) and estimating the proportion of those that were complete, duplicated, fragmented, and absent.

We assembled the eight *A. granulata* transcriptomes and the transcriptomes of other chiton tissues listed above with Trinity v. 2.84 (Grabherr et al. 2011), using the –trimmomatic and –normalize reads flags. We ran CD-Hit v. 4.8.1 (Fu et al. 2012) on each transcriptome separately to cluster isoforms. We also generated a composite transcriptome of all of the *A. granulata* tissues (eight total transcriptomes including four separate radula regions) by combining reads and then following the same process described above. We used this composite transcriptome for annotation.

### Genome Annotation

To annotate the *A. granulata* genome, we first generated a custom repeat library with RepeatModeler v. 2.0 (Smit and Hubley 2015), which was used in all subsequent analyses. We trained MAKER v. 2.31.10 (Cantarel et al. 2008) on the composite transcriptome described above as well as predicted protein sequences from the tissues of other species of chitons listed above. Using the highest quality gene models from the first as a maker-input gff3 (AED < 0.5), we ran a second round of MAKER. From these resulting gene models, we used those with an AED < 0.25 to train Augustus v3.0.3 (Stanke et al. 2006): we extracted gene models from the genomic scaffolds along with 1,000 bp of flanking sequence on either side to ensure complete genes, and ran them through BUSCO to produce an Augustus model (.hmm) file. Separately, we ran PASA 2.4.1 (Haas et al. 2003) on our composite transcriptome to maximize mapping transcripts to the genome assembly. We were unable to use Evidence Modeler (EVM; Haas et al. 2008) to combine lines of evidence because high levels of alternative splicing caused EVM to consistently reduce the number of “passing” gene models to under 5000. We instead combined results from PASA and a trained Augustus run using the intersect tool in BEDtools v. 2.29.2 (Quinlan and Hall 2010), which removed identical sequences. This yielded a set of 81,691 gene models. When we ran a BUSCO v. 3.9 analysis (Metazoa odb9 data set), we found a 15.2% duplication rate. To decrease duplications caused by transcripts predicted for the same locus by both Augustus and PASA that varied in length (and thus were not removed by the BEDtools intersect tool), we clustered the first set of gene models using cdhit-EST v. 4.8.1 (Fu et al. 2012), which we ran with the slow-but-accurate (−g) flag and with a cluster threshold value of 0.8. This produced a set of 20,470 genes. All commands we used are available in supplementary appendix 1, Supplementary Material online.

To identify annotated proteins in *A. granulata*, we first used Transdecoder (Douglas 2018) to produce peptide files of predicted proteins. We ran Interproscan on the set of 20,470 genes referenced above to identify GO terms and Pfam matches for proteins where possible. We used GHOSTX in the Kaas pipeline (Moriya et al. 2007) to identify KEGG pathways via comparisons to all the available molluscan taxa (*Lottia gigantea, Pomacea canaliculata, Crassostrea gigas, Mizuhopecten yessoensis*, and *Octopus bimaculoides*). Finally, we looked for shared GO terms between specific taxa with OrthoVenn (Xu et al. 2019), comparing *A. granulata* to *Lottia gigantea, Chrysomallon squaminiferon, Octopus bimaculoides*, and *Crassostrea gigas* (supplementary fig. 13, Supplementary Material online).

### Hox Gene Annotation and Genomic Comparisons

We located the Hox cluster of *A. granulata* by first creating a BLAST database of the *A. granulata* scaffolds and then querying this database with available chiton Hox sequences (Wanninger and Wollesen 2019). We marked *A. granulata* sequences with a BLAST hit at *e*-value 1*e*-8 as potential Hox sequences. We found one clear match for each previously identified chiton Hox gene, all in a single cluster within one scaffold. To verify the absence of *Post1*, we queried the *A. granulata* database with *Post1* sequences from five other molluscs (Wanninger and Wollesen 2019). All matched with low support to the existing *A. granulata Post2* sequence, so we concluded that *Post1* is absent from the *A. granulata* genome assembly. We further verified the absence by querying the *A. granulata* transcriptomes from different tissues for *Post1* sequences. A *Post1* query returned hits that all matched already-annotated Hox sequences, with the best hit matching *Post2*.

To graphically examine synteny between *A. granulata* and other molluscan genome assemblies, we loaded each assembly and annotation into the online COGE SynMap2 (Haug-Baltzell et al. 2017) server and compared *A. granulata* to eight other annotated genomes with default SynMap2 settings. These default settings are strict; to note two regions as syntenic, 5 or more genes must share an order with fewer than 20 additional genes between them. We exported dotplots for each pair of genomes to visualize syntenic regions (or lack thereof), where dots then represent shared regions of five or more orthologous genes without major changes in surrounding gene content. Scaffolds in each dotplot were sorted by length, but differing assembly qualities made some dotplots difficult to read due to a high number of very small scaffolds. We assessed heterozygosity of several molluscan genomes and *A. granulata* by downloading raw paired-end data when possible and using GenomeScope2 online (Vurture et al. 2017). We began with a k-mer of 21 for all taxa; if this model failed to converge, or if the homozygous and heterozygous peaks collapsed in the model, we re-ran the analysis with k-mer values of 17, 19, and 31. One taxon (*Lottia*) did not produce separate peaks at any k-mer value; for all others, we selected the optimal k-mer size based on the best-fitting model (minimizing error percentage).

To permit direct comparisons of repeat content within *A. granulata* and other molluscs, we ran RepeatModeler v2.2 (Smit and Hubley 2015) on the scaffolds of a subset of genome assemblies and *A. granulata.* We used the same default parameters for each run and quantified the number of elements in each repeat family identified by RepeatModeler for each genome assembly we analyzed (LINEs, SINEs, etc.).

### Orthology Inference

To identify orthologous genes shared between *A. granulata* and other molluscs, we used OrthoFinder v. 2.3.7 (Emms and Kelly 2015). We analyzed three separate sets of data: 1) *A. granulata* and genomes of 19 other lophotrochozoans, including 14 other molluscs, 2 annelids, 1 brachiopod, 1 phoronid, and 1 nemertean; 2) *A. granulata*, a subset of the above molluscan genomes, and *Lingula anatina* for detailed comparisons of biomineralization genes and; 3) *A. granulata* and an expanded set of data including both genomes and transcriptomes, including several transcriptomes from aculiferans other than *A. granulata*. For all three analyses we used the unclustered 81,691 gene set for *A. granulata*, knowing that duplicated gene models would cluster together. We removed sequences from our orthogroups that were identical to longer sequences where they overlapped, as well as fragmented sequences shorter than 100 amino acids. We retained orthogroups that had a minimum of four taxa, aligned the sequences within them with MAFFT (Katoh et al. 2002), and cleaned mistranslated regions with HmmCleaner (Di Franco et al. 2019). We used AlignmentCompare (https://github.com/kmkocot/basal_metazoan_phylogenomics_scripts_01-2015; last accessed December 29, 2020) to delete sequences that did not overlap with all other sequences by at least 20 AAs (starting with the shortest sequence meeting this criterion).

### Phylogenetic Analyses

For species tree reconstruction, in cases where two or more sequences were present for any taxon in a single-gene alignment, we used PhyloPyPruner 0.9.5 (https://pypi.org/project/phylopypruner/; last accessed December 29, 2020) to reduce the alignment to a set of strict orthologs. This tool uses single-gene trees to screen putative orthogroups for paralogy. To build single-gene trees based on orthologs, we trimmed alignments with BMGE v1.12.2 (Criscuolo and Gribaldo 2010) and constructed approximately maximum likelihood trees for each alignment with FastTree2 (Price et al. 2010) using the “slow” and “gamma” options. We then used these alignments in PhyloPyPruner with the following settings: –min-len 100 –min-support 0.75 –mask pdist –trim-lb 3 –trim-divergent 0.75 –min-pdist 0.01 –trim-freq-paralogs 3 –prune MI. For data sets 1 (“genomes”) and 3 (“all_taxa”), only orthogroups sampled for at least 85% of the total number of taxa were retained for concatenation. For data set 2 (“biomin_subset”), only orthogroups sampled for all eight taxa were retained. Phylogenetic analyses were conducted on the supermatrix produced by PhyloPyPruner v. 1.0 in IQ-TREE v. 1.6.12 (Nguyen et al. 2015) using the PMSF model (Wang et al. 2018) with a guide tree based on the LG model. Topological support was assessed with 1,000 rapid bootstraps.

### Screening for Known Biomineralization Genes

To identify biomineralization genes in the chiton genome, we began by running OrthoFinder v. 2.3.7 on the gene models of select genomes (corresponding to taxon set #2 described in the Orthology inference section above): *Lingula anatina, Pinctada fucata, Lottia gigantea, Haliotis rufescenes, Scapharca broughtonii, Bathymodiolus platifrons*, and *Acanthopleura granulata.* Once we obtained this set of orthogroups, we created a local BLAST protein database from the orthogroups and queried it for known biomineralization genes. We used a previously identified protein sequence for each gene of interest from NCBI (supplementary table 9, Supplementary Material online) as a query and set an *e*-value cutoff of 1*e*-8 to identify the OrthoFinder orthogroup(s) that contain that biomineralization gene of interest. We aligned the putative matching orthogroup, the query sequence for the gene of interest, and the sequences of other orthogroups that were returned as hits by our blast search with MAFFT with the default settings. We then constructed a phylogenetic tree in IQ-TREE 2 v.1.3.11.1 to verify that the query sequence (the known protein sequence) fell within the orthogroup identified rather than within an orthogroup with a similar protein sequence due to conserved domains.

In the specific case of the PIF protein, to distinguish between PIF and PIF-like proteins in *A. granulata*, we supplemented the OrthoFinder orthogroup including PIF from *Pinctada fucata* with other published PIF sequences from *Hyriopsis cumingii, Mytilus coruscus, Pinctada maxima, Pinctada margaritifera*, and *Pteria penguin*, as well as the *L. stagnalis* PIF-like protein sequence. We aligned the sequences with Mafft with default settings and constructed a phylogenetic tree in IQ-TREE 2 v 1.3.11.1.

Silk-like proteins share similar amino acid composition throughout Metazoa, but the genes that code for them are difficult to identify in genomes because their highly repetitive sequences are often missed by traditional gene annotation tools (McDougall et al. 2016). We looked for silk-like proteins with SilkSlider (McDougall et al. 2016), run with default settings but using SignalP v. 4.01 (Nielsen 2017), which identifies potential silk-like proteins by locating low-complexity repetitive domains and signal peptides. The 31 proteins identified as silk-like by SilkSlider were then uploaded to the SignalP 5.0 webserver (Almagro Armenteros et al. 2019) for further predictions of signal peptides associated with extracellular localization.

To locate and quantify IREs, we screened the 20,470-gene *A. granulata* gene model set using the SIREs 2.0 (Campillos et al. 2010) web server. We also ran SIREs on the subset of genomes used for biomineralization analyses (see OrthoFinder above) for comparison. We compensated for differences in annotation methods by first clustering all coding sequences from each genome with CD-Hit-EST (Fu et al. 2012) with a cluster threshold of 0.8 (to match the threshold value we used earlier to reduce redundancy in the annotations of the *A. granulata* genome). We then ran SIREs on each of these sets of predicted transcripts. We only accepted predicted IREs scored as “high quality” according to the SIREs metric (indicating both sequence and structural characteristics of a functional IRE). We pulled chiton genes containing a high quality IRE from the eight different tissue transcriptomes generated for genome annotation and assessed expression by mapping each back to the genome with Salmon v. 0.11.3 (Patro et al. 2017) to generate quantifications of reads per transcript, and running these quantifications through edgeR (Robinson et al. 2010) to account for transcript length (TPM) and permit direct comparisons of gene expression. We also separated 3′ and 5′ IREs by subsetting the high quality IREs based on whether the IRE was located at the beginning or end of the sequence. We made heatmaps with log-transformed data to compensate for outliers in expression levels with R package prettyheatmap (Kolde 2012). We then analyzed the GO terms enriched in the separate sets of 5′- and 3′-containing genes that were highly expressed in the radula with GOrilla (Eden et al. 2009), using the complete protein set as a background data set and the sets of IRE-containing genes as the target list.

## Supplementary Material

Supplementary data are available at *Genome Biology and Evolution* online.

## Supplementary Material

evaa263_Supplementary_Data
