# Supplementary material for: The Iron-Responsive Genome of the Chiton Acanthopleura granulata
Source: Genome Biol Evol. 2020 Dec 15;13(1):evaa263. doi: 10.1093/gbe/evaa263 (PMC7850002; doi:10.1093/gbe/evaa263)
Supplement: evaa263_Supplementary_Data [file evaa263_supplementary_data.pdf]

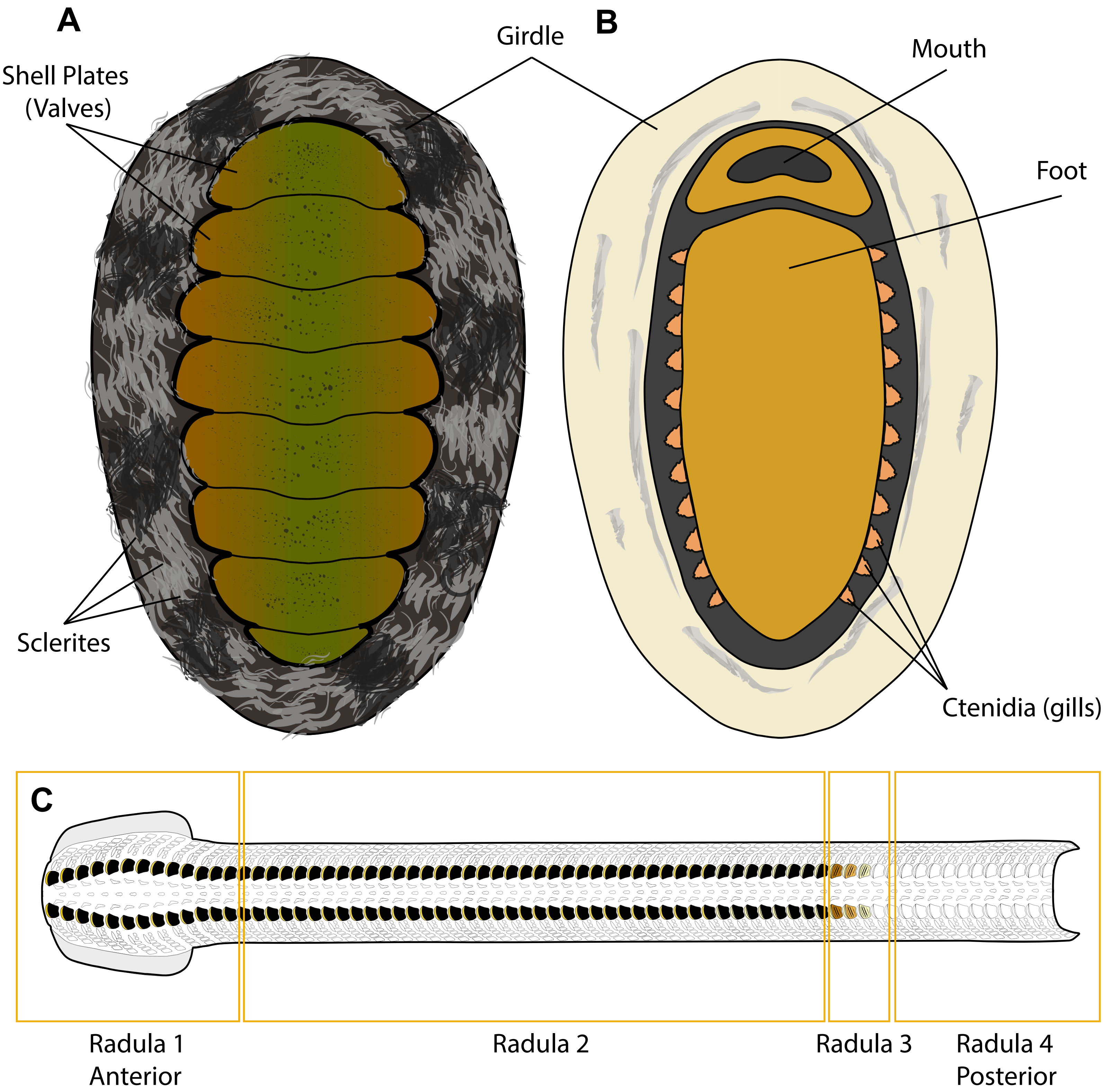

Supplementary Figure 1: An overview of chiton anatomy. **A)** a dorsal view, showing mineralized valves and the sclerites carried on the girdle tissue. **B)** a ventral view, showing the underside of the girdle, mouth, foot, and ctenidia (gills). **C)** A line drawing of the chiton radula with the four pieces referenced throughout the paper numbered anterior-to-posterior. The anterior of the radula is shown to the left.

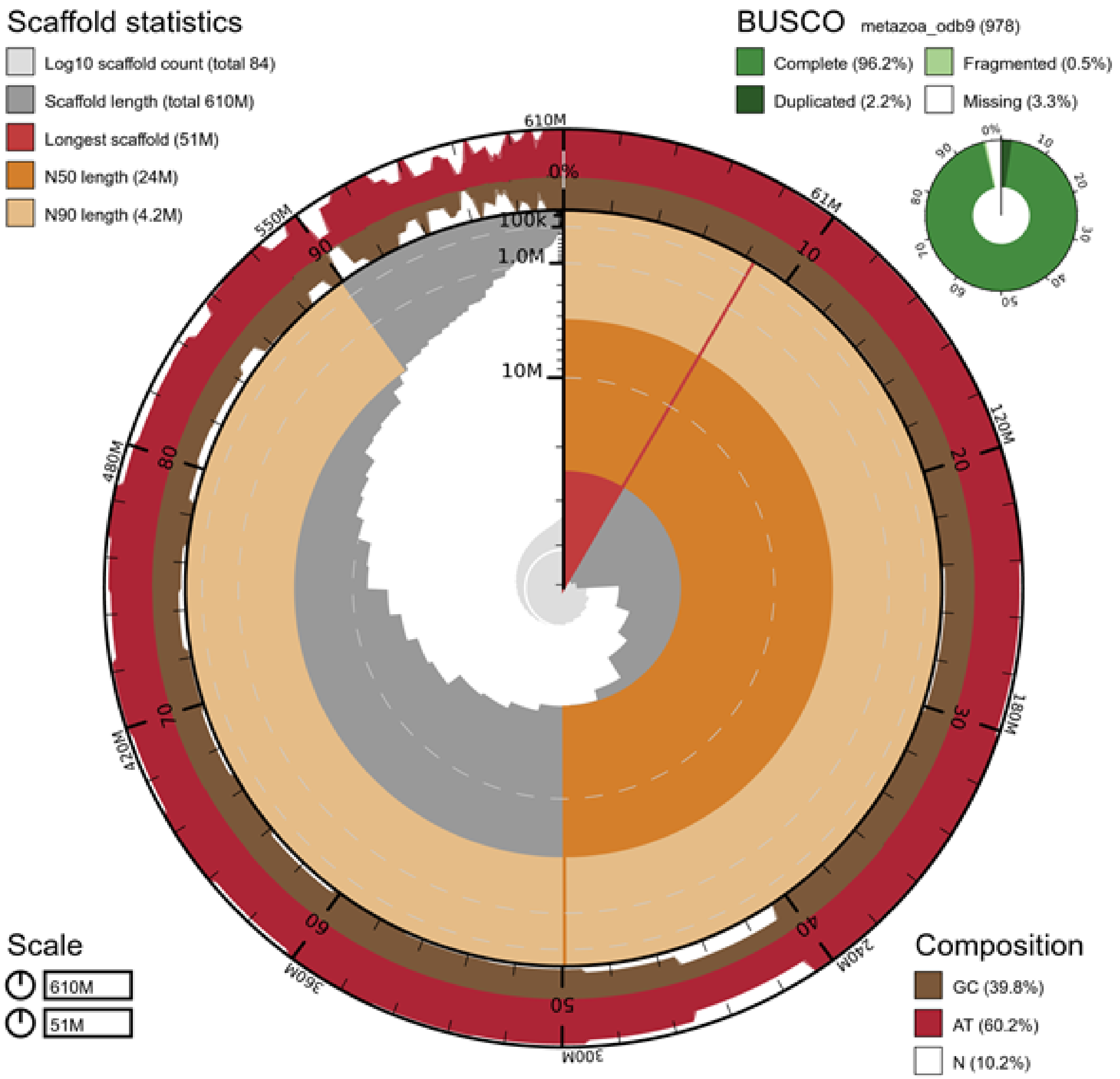

Supplementary Figure 2: Snail plot of the *Acanthopleura granulata* genome assembly. N50 is shown in orange. A preliminary BUSCO analysis (BUSCO v.3, gene set metazoan obd9) in the upper right indicates 96.2% completeness- with updates to BUSCO this improved to 97.4% completeness.

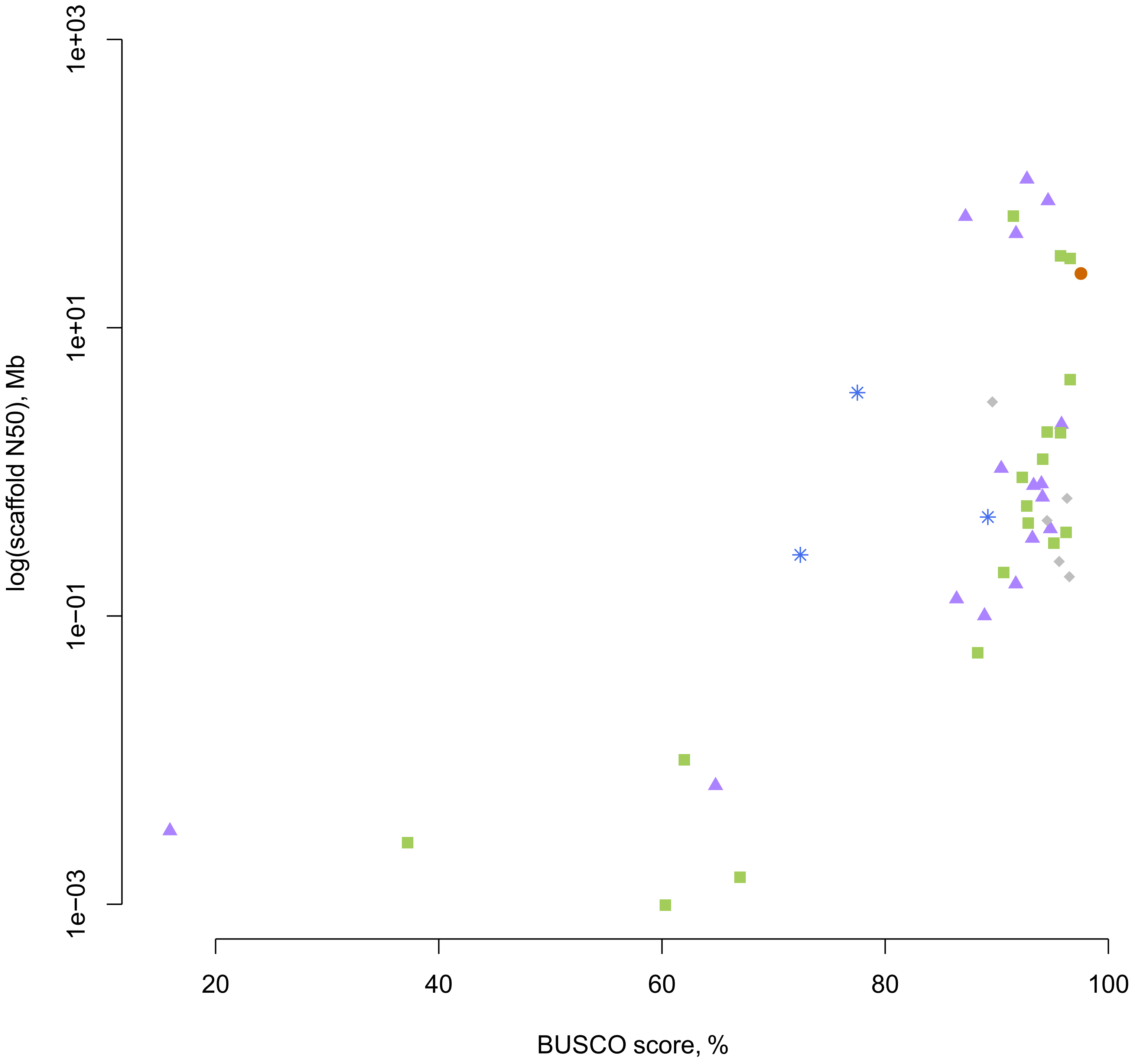

Supplementary Figure 3: An illustration of the quality of available molluscan genomes as a scatter plot of BUSCO completeness score and a log transform of the N50 of scaffolds in megabases for each genome. The *A. granulata* genome is the orange circle, bivalves are purple triangles, gastropods are green squares, and cephalopods are blue stars. Grey diamonds are representative genomes of other lophotrochozoans (specifically those used as outgroups in the homolog gene searches in this study).

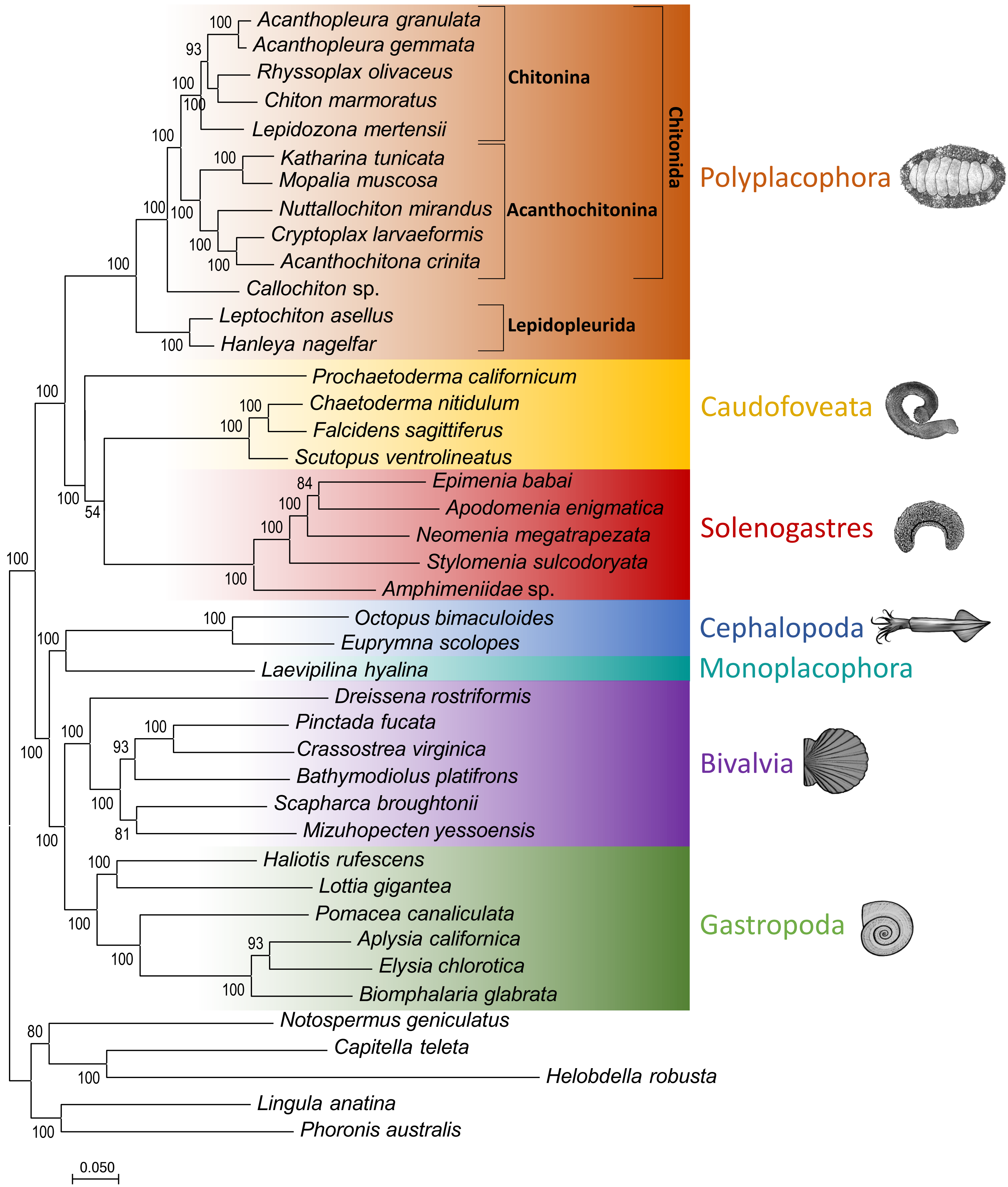

Supplementary Figure 4: An expanded phylogeny based on transcriptome as well as genome sequences, showing the position of A. granulata within a broader range of chitons and other molluscs. Aculifera (Aplacophora [Caudofoveata + Solenogastres] + Polyplacophora) was recovered with maximal support. Within Polyplacophora, A. granulata is recovered within Chitonina, which is also maximally supported.

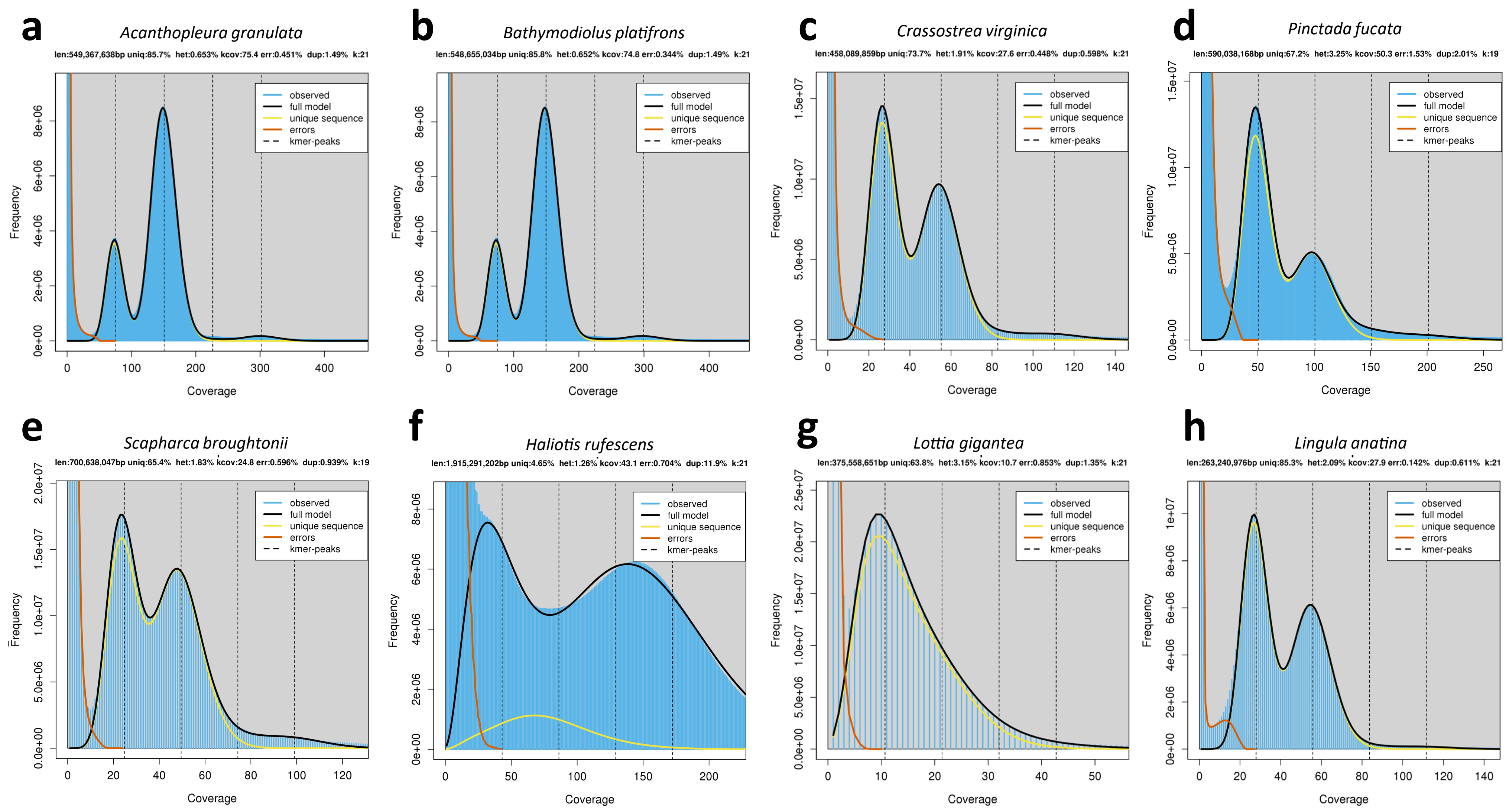

Supplementary Figure 5: GenomeScope analyses of the paired -end data for available molluscan genomes. Heterozygosity is measured via k-mer distribution and available at the top of each graph as het: %. K-mer size is selected from 17, 19, 21, and 31 based on the best-fitting model.

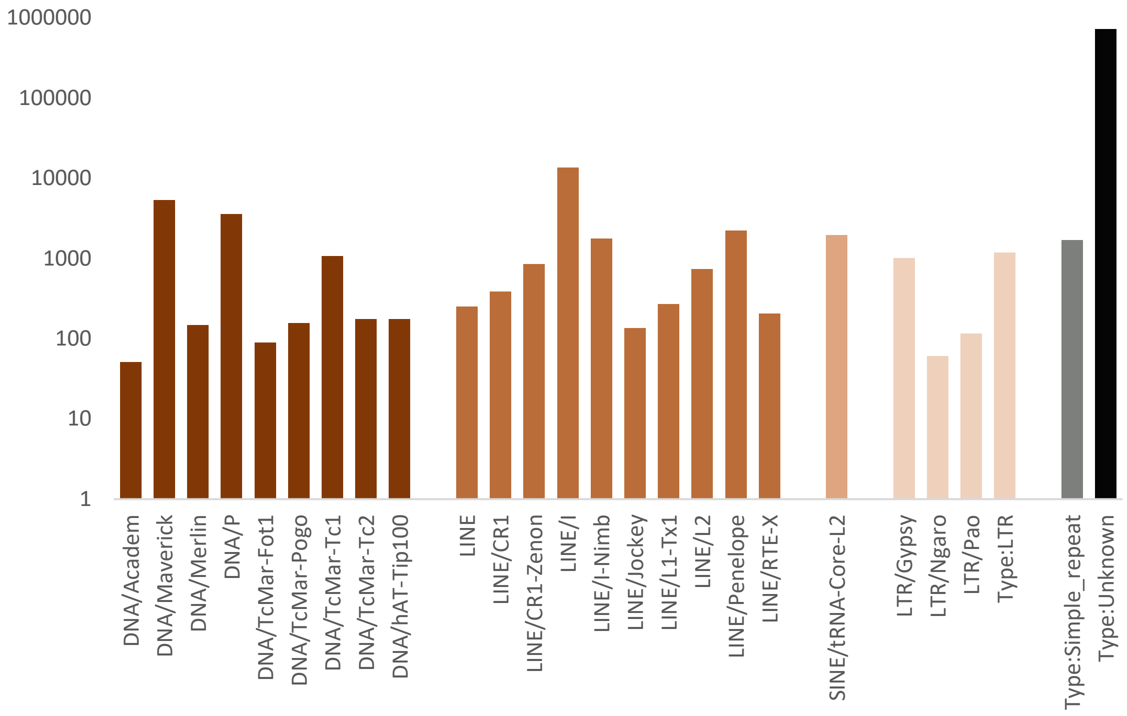

Supplementary Figure 6: Abundance of specific classes of repetitive elements in the *Acanthopleura granulata* genome as quantififed by RepeatModeler.

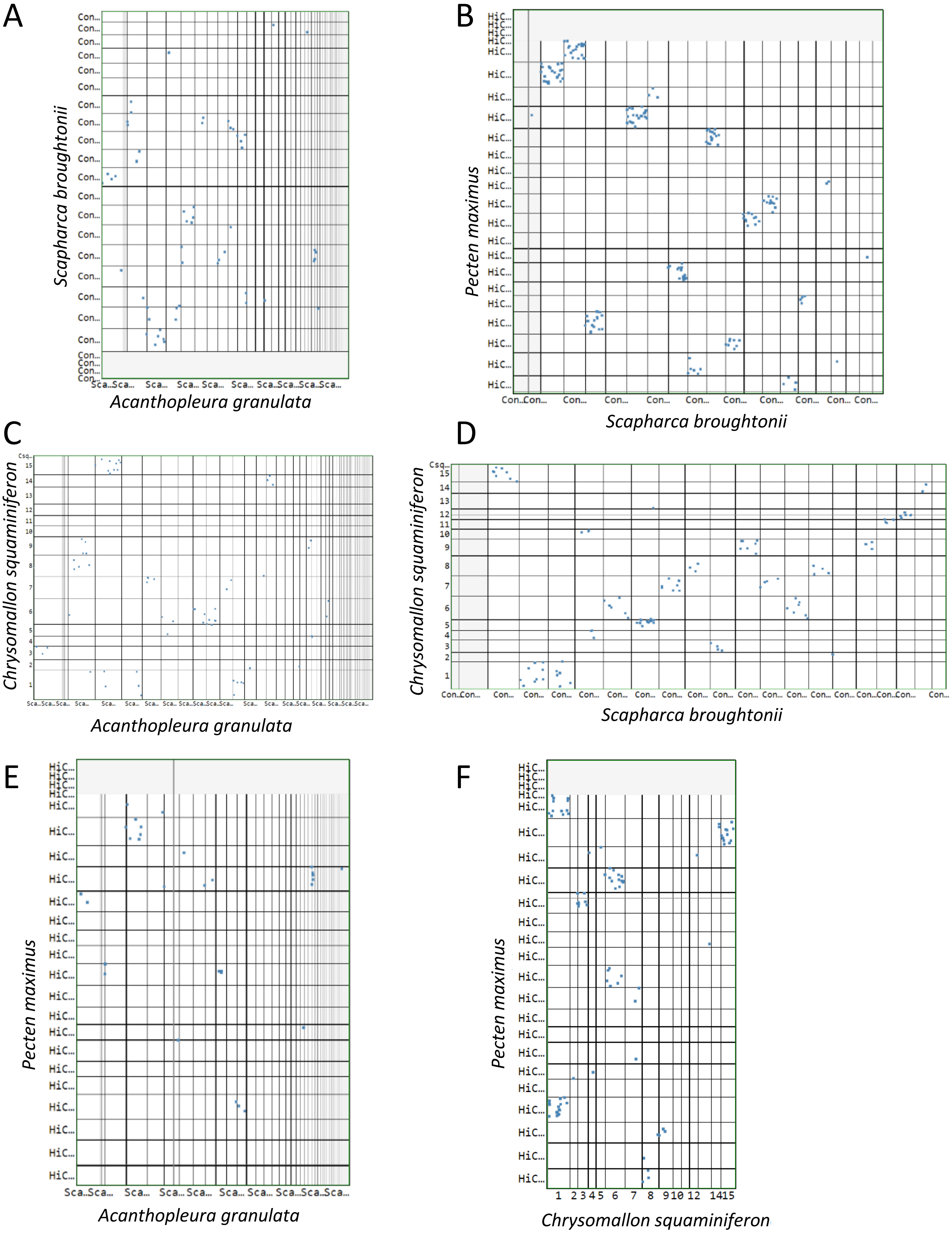
A
Scapharca broughtonii
Acanthopleura granulata
B
Pecten maximus
Scapharca broughtonii
C
Chrysomallon squaminiferon
Acanthopleura granulata
D
Chrysomallon squaminiferon
Scapharca broughtonii
E
Pecten maximus
Acanthopleura granulata
F
Pecten maximus
Chrysomallon squaminiferon

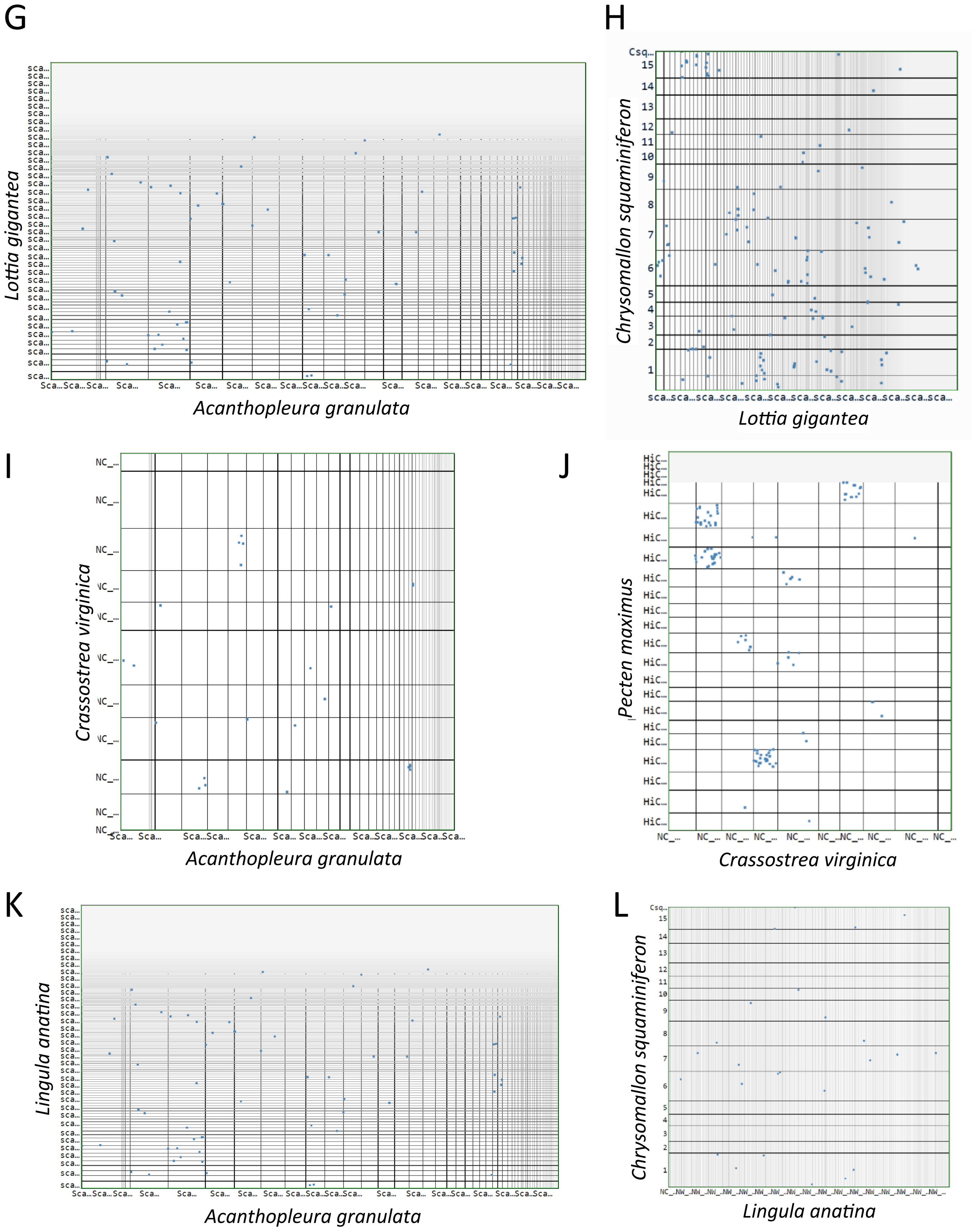

Supplementary Figure 7: Comparisons of synteny between the genome of *Acanthopleura granulata* and other lophotrochozoans, compared to synteny between conchiferans. Blue dots represent regions of synteny as generated in SynMap. Regardless of assembly quality, there is more synteny between two conchiferans (B,D,F,H,J) than between chonchiferans and *A. granulata* (A,C,E,G,I), and *A. granulata* does not share more synteny with a brachiopod than any conchiferan (K-L).

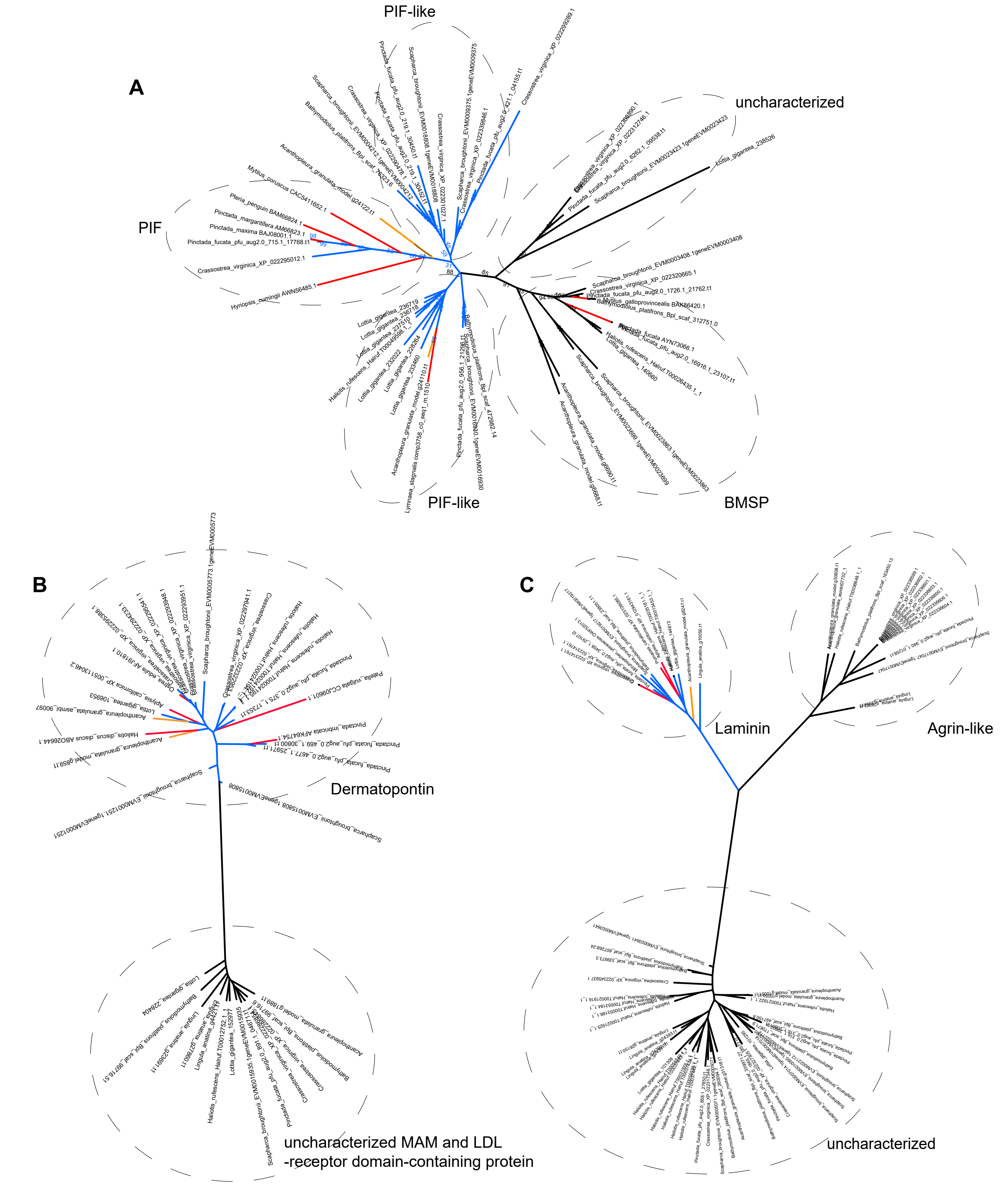

Supplementary Figure 8: Phylogenetic analyses to verify the identification of orthogroups in PIF **(A)**, Dermatopontin **(B)**, and Laminin **(C)**. Orthogroups along with known protein sequences from NCBI were aligned and trees built with IQ-Tree 2. In each tree, the identified orthogroup is shown in blue, the *Acanthopleura granulata* homolog(s) identified in orange, and known protein sequences from NCBI for the gene of interest as well as relevant other orthogroups are shown in red.

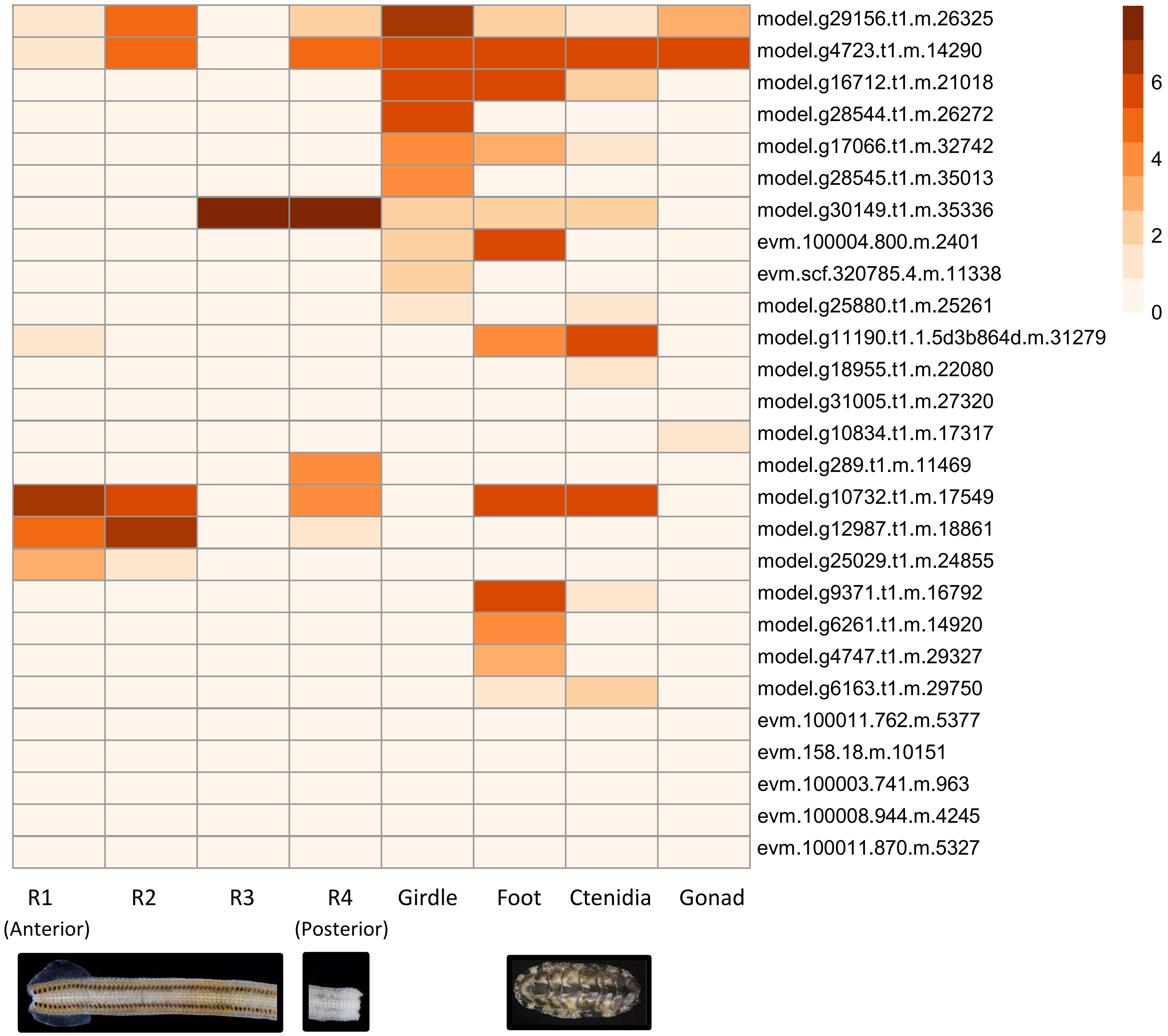

Supplementary Figure 9: Heat map of the expression (log(TPM)) of the 31 genes identified as potentially silk-like by SilkSlider. Expression is higher for several of these genes in the girdle and radula, supporting a potential role of these genes in biomineralization.

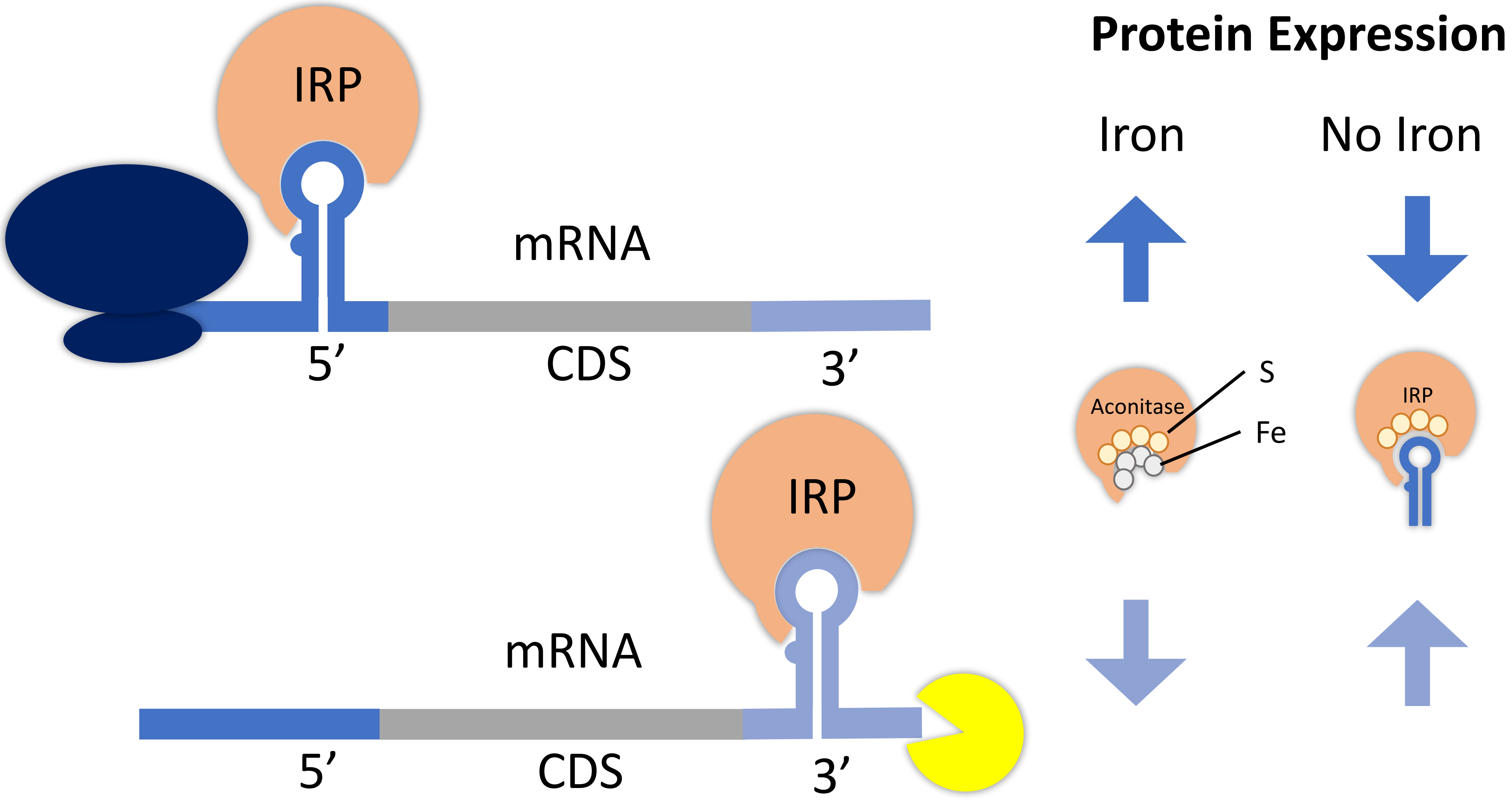

Supplementary Figure 10: The mechanism through which iron response elements (IREs) function. When free iron is present, an IRP will not bind to an IRE because iron (Fe) binds to sulfur (S) at the enzyme’s active site and changes the enzyme’s conformation such that it functions as aconitase (a TCA cycle participant). In the absence of free iron, IRPs bind to IREs, affecting translation rates. When IRPs bind to IREs in the 5’ UTR of an mRNA, they block ribosomes (navy) and prevent translation; thus, mRNAs with 5’ IREs will be translated in the presence of free iron. When IRPs bind to IREs in the 3’ UTR, they block endonucleases (yellow) from degrading mRNA, thereby allowing multiple translations from a single mRNA molecule; thus, the amount of protein produced from mRNAs with 3’ IREs will decrease in the presence of free iron.

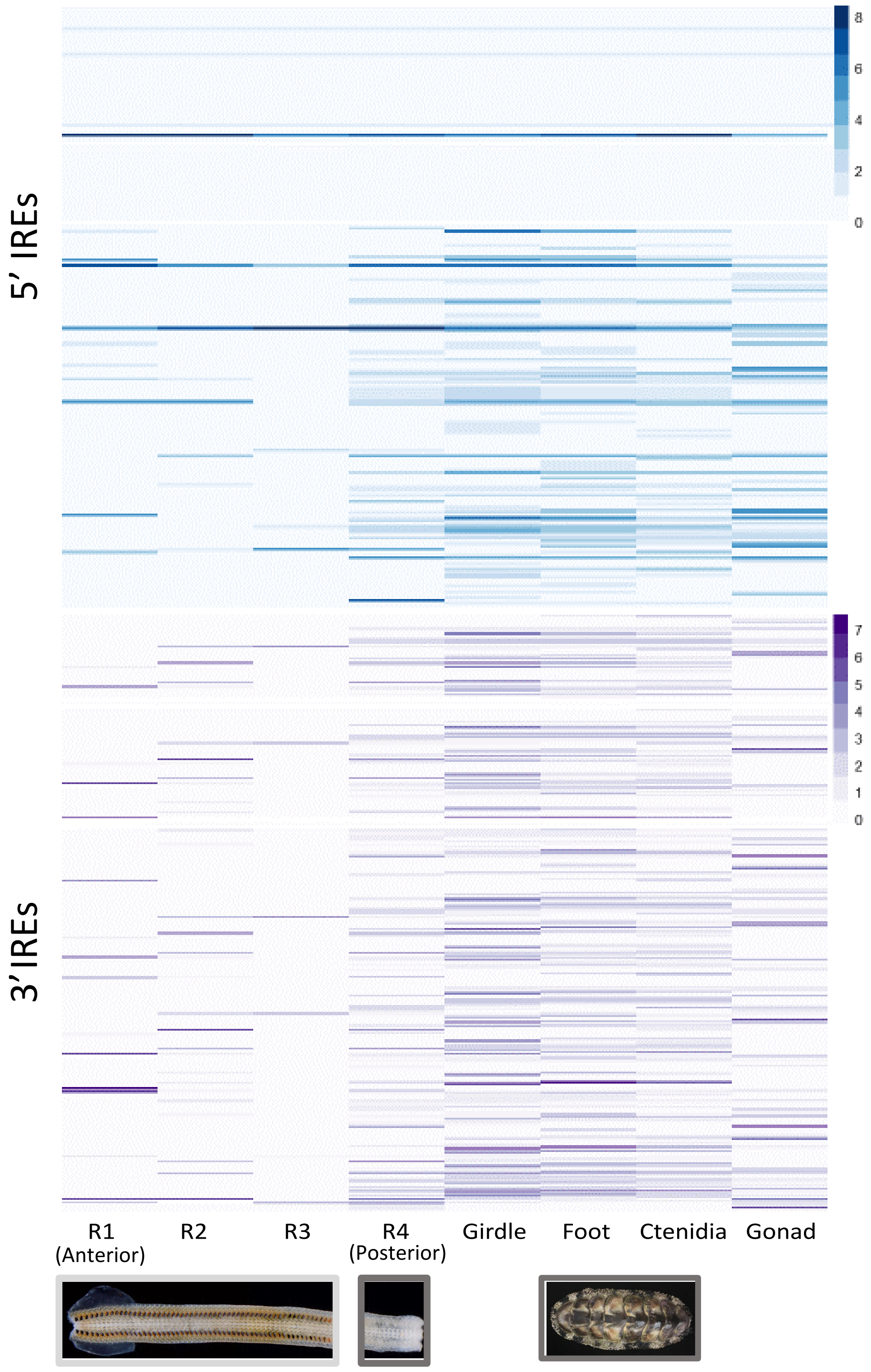

Supplementary Figure 11: The relative expression [log(TPM)] of transcripts containing 5’ IREs (upper, blue) and 3’ IREs in the different tissues of A. granulata. The radula is divided into four developmentally distinct regions: R1,the most anterior region, contains teeth used for feeding; R2 contains teeth that are developed but are not yet used for feeding; R3 contains developing teeth that contain iron oxide; and R4, the most posterior region, contains developing teeth that have yet to be coated with iron. Of the genes that are more highly expressed in the anterior sections of the radula (R1-R3) than in other tissues, we found more 5‘ IRE-containing genes than 3’ IRE-containing genes.

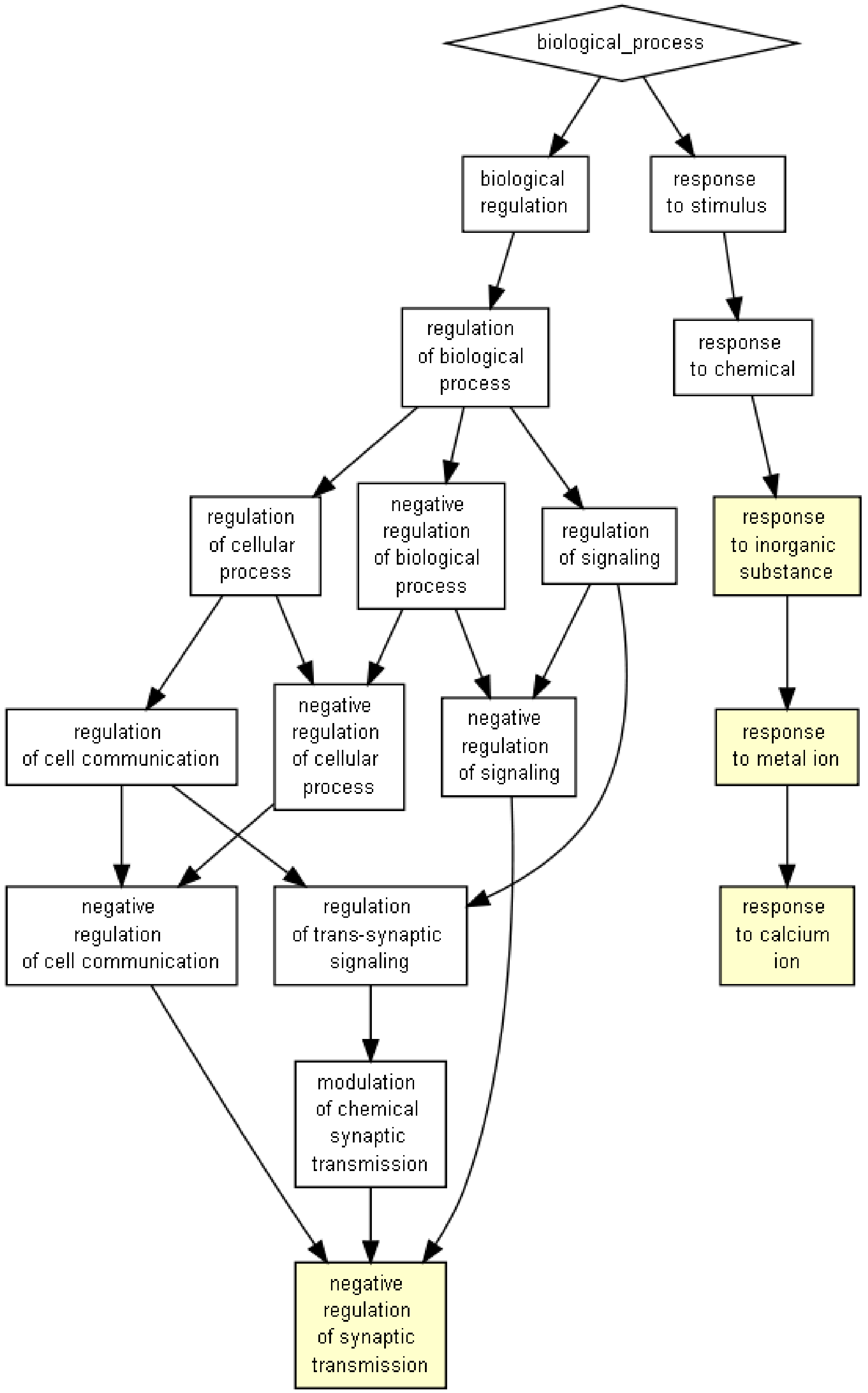

Supplementary Figure 12: GO-term enrichment in the upregulated genes of *Acanthopleura granulata* that contain 5' IREs. Yellow colored boxes indicate biological process GO terms that are enriched in these genes relative to a background gene set of all *A. granulata* genes. The genes that are highly expressed in the radula and have 5'-IREs are enriched for response to inorganic substance, response to metal ion, and response to calcium ion.

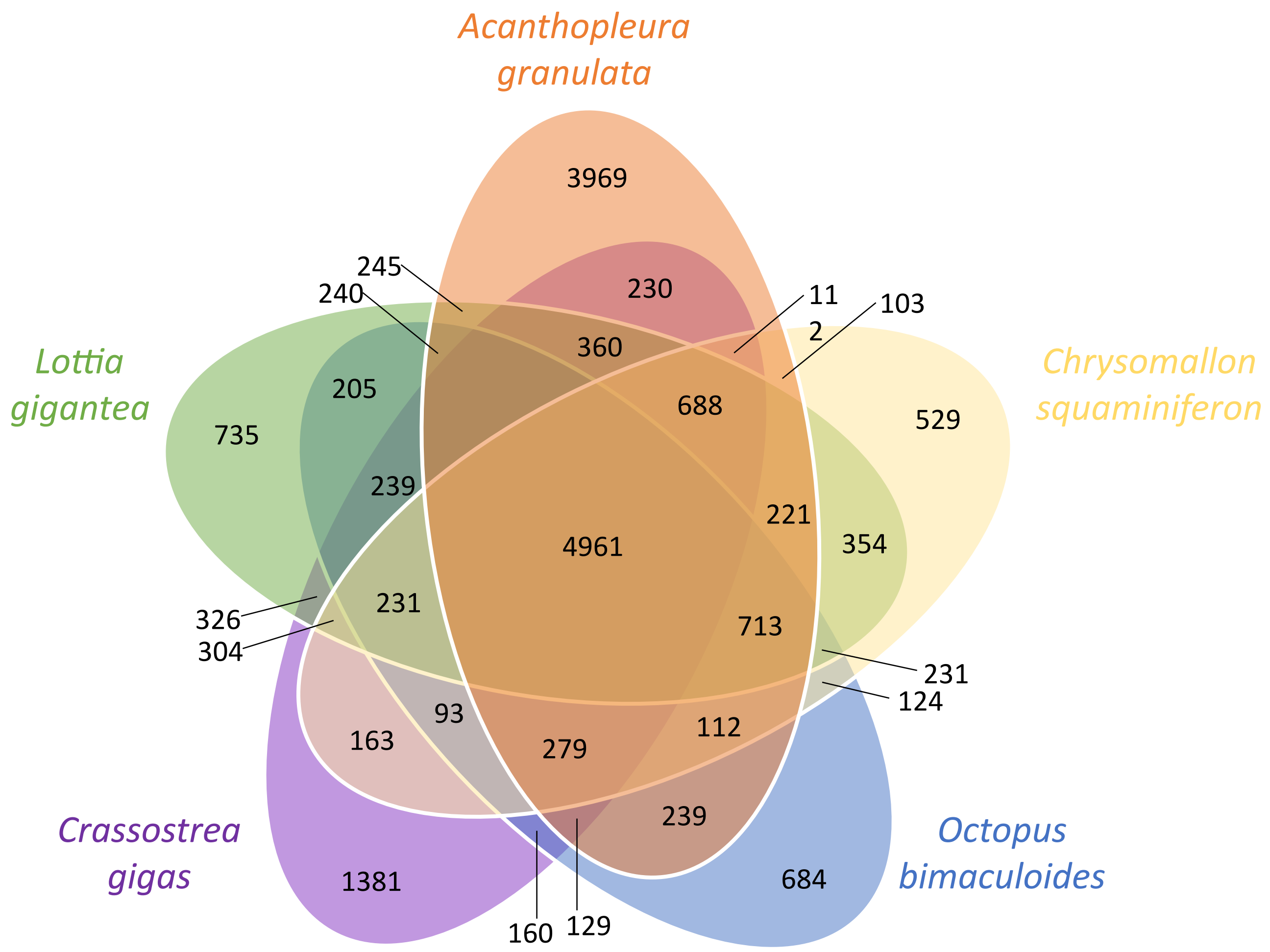

Supplementary Figure 13: Venn diagram of the overlap of orthologous proteins between five molluscs, including *Acanthopleura granulata. A. granulata* has almost as many unique proteins as it shares will all other molluscs present, highlighting the divergenge between *A. granulata* and other sequenced conchiferans.

Supplementary Table 1: Table of molluscan genomes and selected lophotrochozoan genomes available at the time of publication. Details of scaffold continuity were calculated from each genome with QUAST. Completeness scores (BUSCO) were calculated from each genome assembly with BUSCO 3.1, using metazoan gene set obd9. For genomes with available gene models, completeness scores were also calculated with BUSCO 3.1, metazoan gene set obd9, in -transcriptome mode.

Clade	Taxon	Length (Mb)	Number of Scaffolds	N50 (Mb)	Longest scaffold (Mb)	BUSCOgeno(c)	BUSCOtrans(c)	Citation	Source
Polyplacophora	*Acanthopleura granulata*	606	87	24.00	50.9	97.4	96.9	Current study	NCBI PRJNA578131
Cephalopoda	*Euprymna scolopes*	5710	59,146	3.55	29.7	77.5	87.9	Belcaid et al. 2019	NCBI PRJNA470951
Cephalopoda	*Octopus bimaculoides*	2338	151,674	0.49	4.1	89.2	92.8	Albertin et al. 2015	NCBI PRJNA305125
Cephalopoda	*Octopus vulgaris*	1349	77,681	0.27	3	72.4	none	Zarrella et al 2019	NCBI PRJNA492973
Bivalvia	*Argopecten pupuratus*	708	89,727	1.06	11.13	90.4	90.9	Li et al 2018	http://gigadb.org/dataset/view/id/100419
Bivalvia	*Bathymodiolus platifrons*	1658	65,664	0.35	2.79	93.2	86.6	Sun et al. 2017	NCBI PRJNA328542
Bivalvia	*Chlamys farreri*	910	388,151	0.67	6.57	94.1	none	Li et al 2017	NCBI PRJNA185465
Bivalvia	*Crassostrea gigas*	558	7,658	0.40	1.96	94.8	83.4	Zhang et al. 2012	NCBI PRJNA276446
Bivalvia	*Crassostrea virginica*	685	11	75.94	104.16	94.6	98.4	Gomez-Chiarri et al 2015	NCBI PRJNA379157
Bivalvia	*Dreissena polymorpha*	1798	144	107.57	190.5	92.7	none	Penarrubia er al 2015	https://zebra_mussel.s3.msi.umn.edu/Dpolyporpha_Assembly.V2.Final_wMito.fasta.gz
Bivalvia	*Dreissena rostriformis*	1241	18,504	0.13	1.15	86.4	81	Calcino et al 2019	https://phaidra.univie.ac.at/view/o:980132
Bivalvia	*Mizuhopecten yessoensis*	988	82,659	0.83	7.5	94	98.7	Wang et al. 2017	NCBI PRJNA390633
Bivalvia	*Modiolus philippinarum*	2630	74,575	0.10	0.715	88.9	73.5	Sun et al. 2017	NCBI PRJNA328544
Bivalvia	*Mytilus galloprovincialis*	1399	1,002,334	0.00	0.06	15.9	4.2	Murgarella et al 2016	NCBI PRJNA262617
Bivalvia	*Pinctada fucata*	815	29,306	0.17	1.26	91.7	79.4	Takeuchi et al. 2016	http://marinegenomics.oist.jp/pinctada_fucata
Bivalvia	*Pinctada imbricata*	991	5,039	59.03	104.6	87.2	none	Du et al 2017	NCBI PRJNA283019
Bivalvia	*Scapharca broughtonii*	884	1026	44.99	55.6	91.72	90.6	Bai et al. 2019	NCBI PRJNA521075
Bivalvia	*Venustaconcha ellipsiformis*	1590	371,427	0.01	0.31	64.8	none	Renault 2018	NCBI PRJNA433387
Bivalvia	*Saccostrea glomerata*	784	10,107	0.80	7.15	93.3	91.5	Powel et al 2018	NCBI PRJNA414259; http://soft.bioinfo-minzhao.org/srog/#
Bivalvia	*Lutraria rhynchaena*	544	622	2.14	13.02	95.8	none	Thai et al 2019	NCBI PRJNA548223
Gastropoda	*Aplysia californica*	927	4,332	0.92	0.61	92.3	98.6	Broad Institute	NCBI PRJNA13635
Gastropoda	*Biomphalaria glabrata*	916	331,401	0.06	2.18	88.3	92.3	Adema et al. 2017	NCBI PRJNA290623
Gastropoda	*Conus tribblei*	2160	1,126,156	0.00	0.08	37.2	none	Barghi et al 2016	NCBI PRJNA285711
Gastropoda	*Cumia reticulata*	44	144,469	0.00	0.016	67	none	Modica MV et al 2015	NCBI PRJEB9058
Gastropoda	*Elysia chlorotica*	557	9,989	0.44	2.35	92.8	96.9	Bhattacharya et al 2013	NCBI PRJNA484060
Gastropoda	*Haliotis discus hannai*	1865	80,032	0.20	2.21	90.6	33.6	Nam et al. 2017	NCBI PRJNA317403
Gastropoda	*Haliotis rubra*	1378	2,854	1.23	11.18	94.1	none	Kijas et al 2019	NCBI PRJNA489521
Gastropoda	*Haliotis rufescens*	1498	8,371	1.90	13.19	94.5	none	Masonbrink et al 2019	https://abalone.dbgenome.org/downloads
Gastropoda	*Lottia gigantea*	360	4,475	1.87	9.39	95.7	96.5	Simakov et al. 2012	NCBI PRJNA259762
Gastropoda	*Lymnaea stagnalis*	411	85,244	0.01	0.095	62	none	Davison et al 2017	NCBI PRJEB11470
Gastropoda	*Patella vulgata*	28	29,489	0.00	0.015	60.3	none	Kenny et al 2015	https://ora.ox.ac.uk/objects/uuid:6471e7d4-dd34-4eb3-883f-a5c438f11731
Gastropoda	*Pomacea canaliculata*	440	24	31.53	45.4	95.7	98.7	Liu et al. 2018	NCBI PRJNA523959
Gastropoda	*Radix auricularia*	910	4,823	0.58	2.97	92.7	87.2	Schell et al. 2017	NCBI PRJNA350764
Gastropoda	*Chrysomallon squamiferum*	444	22	30.20	49.22	96.6	87.5	Sun et al. 2020	NCBI PRJNA523462
Gastropoda	*Achatina fulica*	1856	921	59.59	116.56	91.5	95.5	Guo et al 2019	http://dx.doi.org/10.5524/100647
Gastropoda	*Lanistes nyassanus*	510	9,826	0.32	1.78	95.1	94.6	Sun et al 2019	NCBI PRJNA523095
Gastropoda	*Marisa cornuarietis*	535	665	4.36	24.07	96.6	95.1	Sun et al 2019	NCBI PRJNA445755
Gastropoda	*Pomacea maculata*	432	3,908	0.38	2.52	96.2	95.7	Sun et al 2019	NCBI PRJNA523958
Brachiopoda	*Lingula anatina*	406	2,677	0.46	2.17	94.5	97.5	Gerdol et al. 2018	NCBI PRJNA293030
Nemertea	*Notospermus geniculatus*	859	11,108	0.24	1.58	95.6	94.5	Luo et al. 2018	NCBI PRJNA393252
Phoronida	*Phoronis australis*	498	3,984	0.66	4.87	96.3	97.2	Luo et al. 2018	NCBI PRJNA393252
Annelida	*Capitella telata*	334	21,042	0.19	1.62	96.5	97.2	Simakov et al. 2012	NCBI PRJNA175705
Annelida	*Helobdella robusta*	235	1,993	3.06	13.6	89.6	92	Simakov et al. 2012	NCBI PFJNA259764

Supplementary Table 2: Comparison of heterozygosity, reproductive mode, and larval stage(s) across taxa for which short read data were available for heterozygosity analysis.

Species	Heterozygosity	Reproductive Mode	Larval Feeding	NCBI Accession
Acanthopleura granulata	0.653	Broadcast spawner (Glynn 1970)	Lecithotrophic	Present study
Bathymodiolus platifrons	0.652	Broadcast spawner, potentially annual (Xu et al. 2018)	Planktotrophic	SRR3866232
Scapharca broughtonii	3.03	Broadcast spawner, annual (Park et al. 2001, Wang et al. 2017)	Planktotrophic	SRR8535392
Crassostrea virginica	1.91	Broadcast spawner, twice annually (Hayes et al. 1980)	Planktotrophic	SRR6159074
Haliotis rufescens	1.11	Broadcast spawner, no clear seasonality (Giorgi et al. 1977)	Planktotrophic	SRR6761051
Pinctada fucata	1.03	Broadcast spawner, annual (Behzadi 1977)	Planktotrophic	DRR001602
Lottia gigantea	3.15	Broadcast spawner (Kay et al. 2005)	Lecithotrophic	SRR097541

Supplementary Table 3: Output from a RepeatModeler + RepeatMasker run on the complete *Acanthopleura granulata* genome, showing the differnt numbers of repeat classes and specific elements, as well as the total percentage of repeats in the genome.

**elements**	**# elements**	**length (bp)**	**% sequence**
SINEs:	1962	137438	0.02
ALUs	0	0	0
MIRs	0	0	0
LINEs:	20660	8934516	1.47
LINE1	0	0	0
LINE2	743	391158	0.06
L3/CR1	453	324545	0.05
LTR elements:	1245	1461746	0.24
ERVL	0	0	0
ERVL-MaLRs	0	0	0
ERV_classI	0	0	0
ERV_classII	0	0	0
DNA elements:	11721	5129973	0.85
hAT-Charlie	0	0	0
TcMar-Tigger	0	0	0
Unclassified:	680821	127239796	20.98
Total interspersed repeats		142903469	23.56

Supplementary Table 4: Previously identified molluscan biomineralization genes that generated orthogroups in our analyses. For each gene, the number of orthogroups is reported, as well as the total number of gene models for each taxon across all orthogroups.

Gene	Type	Function	Reference	Orthogroup(s)	BMOD	SBRO	PFUC	CVIR	LGIG	HRUF	AGRA	LANA
Ferritins	Iron	Iron binding and transport. Secreted subunits may have an immune effect.	Zhang et al 2003	OG0002129, OG0016057	1	4	2	3	3	2	2	2
Ferrochelatase	Iron	May have a role in shell pigmentation.	Song et al 2019	OG0004417	1	1	1	2	1	1	1	1
Haemocyanin	Iron	O_2 courier		OG0011679	0	0	1	1	0	3	1	1
Calponin	Matrix	Cross links muscle fibers; may cross link proteins to stabilize shell matrix	Sleight et al 2016	OG0014997	1	1	1	1	1	1	1	0
Cartilage Matrix Protein	Matrix	Binds calcium crystals and forms part of extracellular matrix	Wang et al. 2016	OG0001343	2	3	3	3	1	6	4	0
Chitin Deacetylase	Matrix	Can produce chitosan from chitin.	Wang et al 2012	OG0002391,OG0002636, OG0010102, OG0012893, OG0015720	5	8	5	5	5	6	9	3
Chitin Synthase	Matrix	Synthesizes chitins, characterized by transmembrane domains in invertebrates	Weiss et al 2006	OG0013488, OG0012243, OG0003820, OG0001957, OG0001889, OG0001819, OG0001528	31	25	31	139	28	44	29	35
Chitinase	Matrix	Destruction and thus guidance of remodeling of chitin	Wang et al 2012	OG0000213, OG0011850, OG0012263, OG0014939, OG0020247	12	12	11	6	9	17	4	8
Collagens	Matrix	Structure, and may guide SMPs	Dyachuk et al 2018, Gao et al 2015, Song et al 2019	OG000476, OG0001343, OG0003241, OG0004451, OG0004905, OG0006935, OG0010555, OG0012231, OG0013112, OG001413, OG0019628	4	6	4	9	7	4	3	6
Dermatopontin	Matrix	ECM protein. Homologs known from 5 mammals and invertebrates. Known to be part of shell matrix.	Zhang et al 2006	OG0001463	0	3	3	7	1	3	2	0
Fibronectin	Matrix	Binds integrins (membrance spanning receptor proteins). Characterized from nacreous layer, mosaic pattern of loss	Marie et al 2011	OG0000914	1	1	1	13	1	1	0	1
Filamin	Matrix	Actin cross-linking	Liao et al 2015	OG0002841	1	1	1	5	1	1	1	1
Laminin	Matrix	Binds to cells and mediates attachment and organization	Aguilera et al 2017	OG0006743	1	1	1	1	1	4	1	1
Papilin	Matrix	Extracellular matrix (similar proteins exist in arthopods) glycoprotein containing several KUNTIZ domains	Gardner et al 2011	OG0003173	5	7	6	34	4	7	10	13
Perlustrin	Matrix	Similar to lustrin A. Contains growth factor receptors (GFR), but only known from Haliotis and Lottia.	Weiss et al 2000	OG0014184	0	2	1	0	0	1	0	0
Vitellogenin	Matrix	lipid transporter		OG0011518	2	1	1	11	0	1	1	1
BMSP	Mineralization	Binds calcium carbonate crystals. Can bind calcite and aragonite. Is preprotein that breaks into BMSP120 (4 VWAs, 1 chitin-binding domain) and BMSP100 (phosporylated and part of nacreous layer, dense in myostracum). Homologous to PIF177	Suzki et al 2011	OG0003624	1	3	2	1	1	1	2	0
Calcitonin Receptor	Mineralization	GPCR that binds calcitonin (peptide hormone) and maintains calcium homeostasis		OG0002433	1	3	3	1	1	1	2	1
Follistatin	Mineralization	Chitin binding, protein interactions	Feng et al 2017	OG0010890	1	1	1	1	1	1	1	1
Nacrein	Mineralization	Synthesizes HCO_3^-	Miyamoto et al 2005	OG0001347	0	0	0	0	1	1	2	2
Perlucin	Mineralization	Increases precipitation of calcium carbonate. Contains C-type lectin domain.	Weiss et al 2000	OG0004979, OG0006217	2	2	4	6	4	4	0	0
Peroxidases	Mineralization	May stimulate collagen production	Feng et al 2017, DeNichilo et al 2016	OG0000097,OG0001338, OG0003933,OG0006494,OG0011251	14	15	13	21	4	17	13	8
PIF80/97 Precursor	Mineralization	Binds to chitin, implicated in crystal growth patterns	Suzuki et al 2013	OG0000555, OG0003624	3	7	7	6	6	1	1	0
Secreted protein acidic and rich in Cysteine (SPARC)	Mineralization	Calciium binding, stabilizes vaterite to inhibit calcite formation (can facilitate nacre formation)	Xie et al 2016	OG0006587	1	1	1	3	1	0	1	1
Tyrosinase	Mineralization	Crosslinks o-diphenols and aids in formation of the periostracal layer	Mann et al 2012	OG0000131	16	18	6	13	1	1	3	7
Carbonic Anhydrases	Mineralization	Interconversion of water and CO_2 to carbonic acid, protons, and bicarbonate ions.	Duvail et al 1997	OG0000554,OG0001347,OG0001347,OG0015230	10	10	9	17	8	10	4	6
Activator protein-1 (AP-1)	Trans. Factor	Binds directly to KRMP and enhances promoter activity	Zheng et al 2015	OG0008602	1	1	1	1	1	1	1	1
Pf-POU3F4	Trans. Factor		Gao et al 2016	OG0002372	1	1	1	1	4	1	1	1
Rel	Trans. Factor	Binds directly to Nacrein and enhances promoter activity	Sun et al 2015	OG0011250	1	1	1	1	1	1	1	1

Supplementary Table 5: Expression (TPM) of the homologs of PIF protein as identified by orthology inference. Expression is shown across the eight *A. granulata* transcriptomes generated in this study. PIF is expressed mainly in the girdle tissue.

PIF homolog	Radula 1 (anterior)	Radula 2	Radula 2	Radula 4 (posterior)	Girdle	Ctenidia	Foot	Gonad
model.g24122.t1	0	0	0	0	37.28773	0	0.096919	0
model.g24110.t1	0	0	0	0.694748	14.57598	0	0	0

Supplementary Table 6: Translated gene models identified by SilkSlider as putative genes for proteins with silk-like domains. A top blastp hit is listed for each protein, along with the name/gene/accession of the most similar result and E-value of the blastp hit. The final column is a separate analysis via SignalP-5.0 of whether a signal peptide is predicted for this gene model (Likelihood % in parentheses).

Gene model	Top blastp hit	Taxon	Accession	Evalue	Signal Peptide Predicted
>evm.model.scf7180000027668.320785.4|m.11338	carbonic anhydrase 14-like	*Crassostrea virginica*	XP_022339696.1	6.00E-56	Yes, 0.9555
>model.g30149.t1|m.35336	chitinase (acidic mammalian)	*Mizuhopecten yessoensis*	OWF47140.1	5.00E-06	Yes, 0.9797
>evm.model.Super-Scaffold_100008.944|m.4245	collagen alpha-2 chain-like	*Acanthaster planci*	XP_022105828.1	6.00E-96	Yes, 0.7671
>model.g17066.t1|m.32742	collagen alpha-2 chain-like	*Mizuhopecten yessoensis*	XP_021346726.1	0.00E+00	Yes, 0.9980
>model.g4747.t1|m.29327	complement C1g subcomponent B-like	*Octopus vulgaris*	XP_029636373.1	3.00E-94	Yes, 0.8324
>model.g9371.t1|m.16792	deodyribonuclease-1-like	*Mizuhopecten yessoensis*	XP_021351957.1	2.00E-59	Yes, 0.7191
>model.g6261.t1|m.14920	eukaryotic translation initiation factor 3 subunit A-like	*Octopus bimaculoides*	XP_014778366.1	4.00E-26	Yes, 0.7402
>evm.model.Super-Scaffold_100004.800|m.2401	fibril-forming collagen alpha chain-like	*Octopus vulgaris*	XP_029638258.1	0.00E+00	Yes, 0.9996
>model.g6411.t1|m.15452	fibrillar collagen (many annelid hits)	*Riftia pachyptila*	AAF80453.1	2.00E-57	Yes, 0.9994
>model.g10834.t1|m.17317	frizzled-5-like, membrance frizzled	*Crassostrea gigas*	XP_011453204.1	1.00E-69	Yes, 0.9714
>evm.model.Super-Scaffold_100003.741|m.963	Histone-lysine N-methyltransferase PRDM9	*Mizuhopecten yessoensis*	OWF46683.1	7.00E-14	No
>model.g5858.t1.1.5d3b846d|m.29547	IgGFc-binding protein-like	*Crassostrea gigas*	XP_011418167.1	1.00E-105	Yes, 0.9833
>model.g18955.t1|m.22080	myosin-IIIb isoform X2	*Lingula anatina*	XP_013418100.1	0.00E+00	No
>model.g4723.t1|m.14290	nucleolin-like	*Lingula anatina*	XP_013398714.2	5.00E-28	Yes, 0.6206
>model.g25880.t1|m.25261	protein SSXT-like	*Mizuhopecten yessoensis*	XP_021354255.1	2.00E-30	No
>model.g29156.t1|m.26325	sialic acid binding lectin	*Helix pomatia*	ABF00124.1	6.00E-09	Yes, 0.9978
>model.g25029.t1|m.24855	uncharacterized	*Lingula anatina*	XP_013408830.1	4.00E-92	Yes, 0.9609
>model.g6163.t1|m.29750	ptotocadherin Fat 4-like	*Lingula anatina*	XP_013414156.1	9.00E-131	Yes, 0.9933
>model.g10732.t1|m.17549	uncharacterized from several molluscs	*Crassostrea gigas*	XP_011429067.1	2.00E-12	Yes, 0.7249
>evm.model.Super-Scaffold_100007.378|m.3990	uncharacterized PE-PGRS family protein PE	*Diabrotica virgifera*	XP_028143928.1	4.50E+00	Yes, 0.9988
>model.g11190.t1.1.5d3b864d|m.31279	uncharacterized, predicted flocculation (FLO10),mucin5	*Crassostrea gigas*	XP_011424285.1	9.00E-62	Yes, 0.9103
>evm.model.Super-Scaffold_100011.870|m.5327	no hits				Yes, 0.9849
>evm.model.Super-Scaffold_158.18|m.10151	no hits				Yes, 0.9761
>model.g12987.t1|m.18861	no hits				Yes, 0.9938
>model.g14042.t1|m.19238	no hits				Yes, 0.6893
>model.g16712.t1|m.21018	no hits				Yes, 0.8798
>model.g28544.t1|m.26272	no hits				Yes, 0.9868
>model.g28545.t1|m.35013	no hits				Yes, 0.7622
>model.g289.t1|m.11469	no hits				Yes, 0.9815
>model.g31005.t1|m.27320	no hits				No
>evm.model.Super-Scaffold_100011.762|m.5377	no hits				Yes, 0.9959

Supplementary Table 7: Total number of gene models in molluscan genome annotations before and after clustering with CDHit, and the number of high-quality IRE predictions from SIRE for each set of clustered gene models across molluscan taxa.

Taxon	Initial Gene	Clustered Gene	High-quality	NCBI Project Accession or Other
Acanthopleura granulata	81691	20470	271	PRJNA578131
Bathymodiolus platifrons	33584	27449	181	PRJNA328542
Crassostrea virginica	66625	35117	138	PRJNA376014
Haliotis rufescens	57785	48537	72	https://abalone.dbgenome.org/
Lottia gigantea	23851	21739	126	PRJNA175706
Pinctada fucata	31477	29486	137	https://marinegenomics.oist.jp
Scapharca broughtonii	24045	22288	201	PRJNA193911

Supplementary Table 8: Soma-ferritin gene models identfied for the biomineralization taxa

Organism	Soma-Ferritin Gene Model
Acanthopleura granulata	model.g24813.t1
Crassostrea virginica	XP_022294039.1
Haliotis rufescenes	Halruf.T00032967.1_1
Lingula anatina	g23042.t1
Lottis gigantea	183389
Pinctada fucata	pfu_aug2.0_258.1_27123.t1
Scapharca broughtonii	EVM0007656.1geneEVM0007656

Supplementary Table 9: Query sequences used for each biomineralization gene of interest, indicating the species the sequence is from and the NCBI accession number of each.

Biomineralization Gene	Query Species	Accession
Activator protein-1 (AP-1)	*Haliotis discus discus*	ADQ43242.1
Amorphous calcium carbonate binding protein (ACCBP)	*Pinctada fucata*	ABF13208.1
BMSP	*Pinctada fucata*	AYN73066.1
Calcitonin Receptor	*Crassostrea gigas*	QDH43372.1
Calponin	*Mizuhopecten yessoensis*	BAP84555.1
Carbonic Anhydrases	*Mizuhopecten yessoensis*	OWF36341.1
Cartilage Matrix Protein	*Pinctada fucata*	AQN80778.1
Chitin Deacetylase	*Hyriopsis cumingii*	AFO53262.1
Chitin Synthase	*Pinctada fucata*	BAF73720.1
Chitinase	*Octopus vulgaris*	X02571.1
Collagens	*Haliotis rufescens*	AIZ03373.1
C-type Lectins	*Pinctada fucata*	ADX95743.1
Dermatopontin	*Pinctada imbricata*	AFK64754.1
Ferritins	*Mizuhopecten yessoensis]*	AHH31563.1
Ferrochelatase	*Pomacea canaliculata*	XP_025082221.1
Fibronectin	*Mizuhopecten yessoensis*	OWF41565.1
Filamin	*Euprymna scolopes*	AAT99400.1
Follistatin	*Mizuhopecten yessoensis*	OWF54001.1
Haemocyanin	*Aplysia californica*	CAD88977.1
Laminin	*Crassostrea virginica*	XP_022314761.1
Lustrin	*Haliotis tuberculata*	ADM52208.2
Nacrein	*Pinctada fucata*	BAA11940.1
Papilin	*Mizuhopecten yessoensis*	OWF36203.1
Perlucin	*Mizuhopecten yessoensis*	OWF55388.1
Perlustrin	*Mizuhopecten yessoensis*	OWF44587.1
Peroxidases	*Pinctada fucata*	ALK82327.1
Pf-POU3F4	*Pinctada fucata*	AKJ32469.1
PIF80/97 Precursor	*Pinctada margaritifera*	BAM66823.1
Rel	*Mizuhopecten yessoensis*	AKC01670.1
Secreted protein acidic and rich in Cysteine (SPARC)	*Pinctada fucata*	AND99565.1
Tyrosinase	*Mizuhopecten yessoensis*	AKE79095.1
Vitellogenin	*Scapharca broughtonii*	AYE92811.1

Supplementary Document 1: All programs, configuration files, and code used in this study.

**Program**	**Function in present study**	**Version**
Alignment_Compare	Removing sequences that don't meet minimum overlap requirements within orthogroups	1.0
Augustus	Gene prediction	3.3.2
BEDTools	Combining multiple sets of gene predictions	2.29.2
Bionano Solve	Using optical mapping data to scaffold	3.4
Blast+	Identifying orthogroups that matched biomineralization genes	2.10.0
BlobTools	Assessing genome contamination, and generation of snail plot	2.0
BMGE	Trim ambiguously aligned columns from orthogroup alignments	1.12
Bowtie	Mapping PE data back to genome in preparation for PILON	2.2.5
BUSCO	Assessing genome completeness and duplication	4.0.2 / 3.9
CDHit	Markov clustering of transcripts and gene models	4.8.1
EdgeR	Generating TPM values for expression data	3.28.1
EVM	Tools within used for sequence analysis	1.1.1
FastTree	Initial trees within orthogroups to give PhyloPyPruner	2.1
HMMCleaner	Remove sequence regions that are misaligned in orthogroups	0.180750
IQTree	Phylogenetic tree building	1.6.12/1.3.11
MAFFT	Aligning sequences for phylogenetic tree building	7.45
Maker	Annotating genome to generate training files for SNAP and Augustus	2.31.10

MaSuRCA	Hybrid assembly of genome from paired-end and long-read sequencing data	3.3.5
OrthoFinder	Determination of orthogroups between taxa	2.3.8
PASA	Mapping transcriptomic data back to genome assembly to generate an annotation	2.3.3
PhyloPyPruner	Prune paralogs from orthology inferences after OrthoFinder	1.0
PILON	Map paired-end data back to genome assembly and correct potential errors produced by long read sequencing	1.23
PoreChop	Trim nanopore-specific adapter sequences, and discard chimeric sequences	0.2.4
QUAST	Assess genome quality	5.0.2
RaxML	Generate maximum likelihood phylogenies	8.2.12
Reapr	Map paired end data back to final genome assembly to assess completeness	1.0.18
Redundans	Collapse contigs that differ due to heterozygosity in genome assembly	0.14a
RepeatMasker	Generate a file to soft-mask repetitive regions in the genome during annotation	4.0.9/4.1.1
RepeatModeler	Determine the repetitive content of genomes	2.0/2.2
Salmon	Map transcriptome reads back to genome and generate quantifications per isoform	0.11.3
SilkSlider	Identify possible silk-like proteins	0.2.2
SIRE	Identify potential IREs	2.0
SNAP	*De novo* gene prediction	5th update
SynMap (COGE)	Generate dot plots to represent synteny between genomes	2.0

Transdecoder	Identify the longest open reading frame for each gene model and translate to protein sequence	5.5.0
Trinity	Assembly transcriptomes	2.8.4
uniqHaplo.pl	Removing redundant sequences from orthogroups	0.1.4

Genome Assembly:

MaSuRCA:

masurcaconfig.txt file:

```
DATA
#HiSeq X with 2 X 150 bp reads
PE= pe 150 20 AGRA_1.fastq AGRA_2.fastq
NANOPORE=nanoporecombined.fastq
END

PARAMETERS
EXTEND_JUMP_READS=0
GRAPH_KMER_SIZE = auto
USE_LINKING_MATES =1
LIMIT_JUMP_COVERAGE = 300
CA_PARAMETERS = cgwErrorRate=0.15
KMER_COUNT_THRESHOLD = 1
CLOSE_GAPS=1
NUM_THREADS = 16
JF_SIZE = 200000000
SOAP_ASSEMBLY=0
END
```

Quast to evaluate genome assemblies:

```
use quast
quast.py -t 8 --plots-format pdf genome.fasta
```

TrimGalore to trim PE data before mapping:

```
use trim_galore
mkdir trimmed
trim_galore -fastqc --paired AGRA_1.fastq AGRA_2.fastq -o trimmed
```

Bowtie mapping:

```
bowtie2 -p 8 -x scaffolds_bt2_index -1 AGRA_1.fq -2 AGRA_2.fq |
samtools view -Sb - > PE_mapped_for_pilon.corrected.sam
samtools view -bh PE_mapped_for_pilon.corrected.sam -o
PE_mapped_for_pilon.corrected.bam
samtools sort PE_mapped_for_pilon.corrected.bam -o
PE_mapped_for_pilon.corrected.sorted.bam
samtools index PE_mapped_for_pilon.corrected.sorted.bam
samtools depth PE_mapped_for_pilon.corrected.sorted.bam > depth.txt
```

PILON:

```
srun java -Xmx256G -jar /share/apps/bioinfoJava/pilon-1.23.jar --
genome scaffolds.fasta --bam PE_mapped_for_pilon.corrected.sorted.bam
--output AGRA.pilon1.corrected --outdir PILON_output_1 --changes --
diploid  --verbose
```

Redundans:

```
use redundans
redundans.py -t 16 -m 255 --noscaffolding --norearrangements -i
AGRA_1.fastq AGRA_2.fastq -f final.genome.scf.fasta
```

Genome Annotation:

Overall Approach:
Maker was run twice with transcriptome evidence as est2genome and multiple other chitons' protein evidence. Each time, the best-scoring set of proteins +1000 framing basepairs were removed into new files, and used to train Augustus. SNAP was also trained each cycle. The training file produced by the second run was carried forward into Augustus *de novo* predictions. The trained Augustus performed much better (when species=fly BUSCO obd9 complete was 32%, versus with species=agra_round2, 92%).

PASA was run to map transcripts back to the repeat-masked genome, using the composite transcriptome cleaned according to PASA built-in process.

EVM would not work on chitons. Examination of outputs seems to indicate that chitons often alternatively splice exon 1, and EVM tended to discard predictions where this discordance existed.

PASA and Augustus outputs were combined, and bedtools intersect used to remove perfectly redundant models. This left the first set of 81K gene models. CDHit was run to cluster these based on probably redundancy, and at a similarity cutoff of 0.8 produced the 20K gene model set.

Repeat Modeling and Masking:
```
RepeatModeler/BuildDatabase -name AGRA -engine ncbi
/home/scutopus/maker/2018_12_20_AGRA_post_redundans_optical/AGRAredund
ansoptical.fasta
RepeatModeler/RepeatModeler -pa 4 -engine ncbi -database AGRA 2>&1 |
tee repeatmodeler.log
```

Maker Run 1:
Thanks to maker resources at:
http://weatherby.genetics.utah.edu/MAKER/wiki/index.php/The_MAKER_control_files_explained
https://github.com/sujaikumar/assemblage/blob/master/README-annotation.md
https://gist.github.com/darencard/bb1001ac1532dd4225b030cf0cd61ce2

Run to generate a first pass set of gene models. Note est2genome=1 using composite *A. granulata* transcriptome for most complete annotation possible. Protein evidence incorporated from several additional chiton species as listed.

Maker1_opts.ctl file:

```
#-----Genome (these are always required)
##Added genome file here:
genome=scaffolds.fasta
```

```
organism_type=eukaryotic #eukaryotic or prokaryotic. Default is
eukaryotic

#-----Re-annotation Using MAKER Derived GFF3
maker_gff= #MAKER derived GFF3 file
est_pass=0 #use ESTs in maker_gff: 1 = yes, 0 = no
altest_pass=0 #use alternate organism ESTs in maker_gff: 1 = yes, 0 =
no
protein_pass=0 #use protein alignments in maker_gff: 1 = yes, 0 = no
rm_pass=0 #use repeats in maker_gff: 1 = yes, 0 = no
model_pass=0 #use gene models in maker_gff: 1 = yes, 0 = no
pred_pass=0 #use ab-initio predictions in maker_gff: 1 = yes, 0 = no
other_pass=0 #passthrough anything else in maker_gff: 1 = yes, 0 = no

#-----EST Evidence (for best results provide a file for at least one)
est=../Acanthopleura_granulata_KK701_composite_cd-hit.fas #set of ESTs
or assembled mRNA-seq in fasta format
altest= #EST/cDNA sequence file in fasta format from an alternate
organism
est_gff= #aligned ESTs or mRNA-seq from an external GFF3 file
altest_gff= #aligned ESTs from a closely related species in GFF3
format

#-----Protein Homology Evidence (for best results provide a file for
at least one)
protein= Acanthochitona_crinita-
SRR5110525.fa.transdecoder.pep,Acanthopleura_gemmata_contaminant_free_
trinity.fa.transdecoder.pep,Acanthopleura_granulata_KK701_composite_cd
-hit.fa.transdecoder.pep
Callochiton_sp-
Pl49_4C.fa.transdecoder.pep,Chaetopleura_apiculata_girdle.fa.transdeco
der.pep,Chiton_marmoratus-
Speiser.fa.transdecoder.pep,Hanleya_nagelfar_mantle.fa.transdecoder.pe
p,Leptochiton_asellus-
mantle.fa.transdecoder.pep,Mopalia_muscosa_KK364-1-
4R.fa.transdecoder.pep,Tonicella_lineata_KK374-1-
4R.fa.transdecoder.pep #protein sequence file in fasta format (i.e.
from multiple organisms)
protein_gff=  #aligned protein homology evidence from an external GFF3
file

#-----Repeat Masking (leave values blank to skip repeat masking)
model_org=all #select a model organism for RepBase masking in
RepeatMasker
##AGRA repeat library added!
```

```
rmlib=../consensi.fa #provide an organism specific repeat library in
fasta format for RepeatMasker
repeat_protein=/share/apps/maker/data/te_proteins.fasta #provide a
fasta file of transposable element proteins for RepeatRunner
rm_gff= #pre-identified repeat elements from an external GFF3 file
prok_rm=0 #forces MAKER to Repeatmask prokaryotes (no reason to change
this), 1 = yes, 0 = no
softmask=1 #use soft-masking rather than hard-masking in BLAST (i.e.
seg and dust filtering)

#-----Gene Prediction
snaphmm= #SNAP HMM file#SNAP HMM file
gmhmm= #GeneMark HMM file
augustus_species=fly
fgenesh_par_file= #FGENESH parameter file
pred_gff= #ab-initio predictions from an external GFF3 file
model_gff= #annotated gene models from an external GFF3 file
(annotation pass-through)
## Changed to 1 to allow transcriptome to be used directly to annotate
est2genome=1 #infer gene predictions directly from ESTs, 1 = yes, 0 =
no
protein2genome=1 #infer predictions from protein homology, 1 = yes, 0
= no
trna=0 #find tRNAs with tRNAscan, 1 = yes, 0 = no
snoscan_rrna= #rRNA file to have Snoscan find snoRNAs
unmask=1 #also run ab-initio prediction programs on unmasked sequence,
1 = yes, 0 = no

#-----Other Annotation Feature Types (features MAKER doesn't
recognize)
other_gff= #extra features to pass-through to final MAKER generated
GFF3 file

#-----External Application Behavior Options
alt_peptide=C #amino acid used to replace non-standard amino acids in
BLAST databases
cpus=1 #max number of cpus to use in BLAST and RepeatMasker (not for
MPI, leave 1 when using MPI)

#-----MAKER Behavior Options
max_dna_len=100000 #length for dividing up contigs into chunks
(increases/decreases memory usage)
min_contig=1 #skip genome contigs below this length (under 10kb are
often useless)
```

```
pred_flank=200 #flank for extending evidence clusters sent to gene
predictors
pred_stats=0 #report AED and QI statistics for all predictions as well
as models
AED_threshold=1 #Maximum Annotation Edit Distance allowed (bound by 0
and 1)
min_protein=0 #require at least this many amino acids in predicted
proteins
alt_splice=0 #Take extra steps to try and find alternative splicing, 1
= yes, 0 = no
always_complete=0 #extra steps to force start and stop codons, 1 =
yes, 0 = no
map_forward=0 #map names and attributes forward from old GFF3 genes, 1
= yes, 0 = no
keep_preds=0 #Concordance threshold to add unsupported gene prediction
(bound by 0 and 1)

split_hit=10000 #length for the splitting of hits (expected max intron
size for evidence alignments)
single_exon=1 #consider single exon EST evidence when generating
annotations, 1 = yes, 0 = no
single_length=250 #min length required for single exon ESTs if
'single_exon is enabled'
correct_est_fusion=0 #limits use of ESTs in annotation to avoid fusion
genes

tries=2 #number of times to try a contig if there is a failure for
some reason
clean_try=0 #remove all data from previous run before retrying, 1 =
yes, 0 = no
clean_up=0 #removes theVoid directory with individual analysis files,
1 = yes, 0 = no
TMP= #specify a directory other than the system default temporary
directory for temporary files
```

Reformat outputs for training:

```
gff3_merge -s -d maker1_master_datastore_index.log >
maker1.all.maker.gff
fasta_merge -d maker1_master_datastore_index.log
gff3_merge -n -s -d maker1_master_datastore_index.log >
maker1.all.maker.noseq.gff
```

SNAP training:

```
mkdir snap/round2
```

Using an AED cutoff of 0.25 to pull the higher quality models:

```
maker2zff -x 0.25 -l 50 -d ../../maker2_master_datastore_index.log
```
This produces a .ann and a .dna file.
Everything must be renamed carefully or SNAP will fail to find the files later:
```
rename 's/genome/AGRA_rnd2.zff.length50_aed0.25/g' *
```
We now pull these gene models with 1000 basepairs of extra sequence to make sure we get the entire gene:
```
fathom AGRA_rnd2.zff.length50_aed0.25.ann
AGRA_rnd2.zff.length50_aed0.25.dna -gene-stats > gene-stats.log 2>&1
fathom AGRA_rnd2.zff.length50_aed0.25.ann
AGRA_rnd2.zff.length50_aed0.25.dna -validate  > validate.log 2>&1
fathom AGRA_rnd2.zff.length50_aed0.25.ann
AGRA_rnd2.zff.length50_aed0.25.dna -categorize 100 > categorize.log
2>&1
fathom uni.ann uni.dna -export 1000 -plus > uni-plus.log 2>&1
```
Now make a directory for SNAP to find:
```
mkdir params
cd params
forge ../export.ann ../export.dna > ../forge.log 2>&1
cd ..
hmm-assembler.pl AGRA_rnd2.zff.length50_aed0.25 params >
AGRA_rnd2.zff.length50_aed0.25.hmm
```

Augustus Training
```
mkdir augustus
cd augustus
awk -v OFS="\t" '{ if ($3 == "mRNA") print $1, $4, $5 }'
../maker2_rnd2.all.maker.noseq.gff | \
  awk -v OFS="\t" '{ if ($2 < 1000) print $1, "0", $3+1000; else print
$1, $2-1000, $3+1000 }' | \
  bedtools getfasta -fi ../../AGRAredundansoptical.fasta -bed - -fo
maker2_rnd2.all.maker.transcripts1000.fasta
```
We will use Augustus within BUSCO to create the training file:
```
BUSCO.py -i maker2_rnd2.all.maker.transcripts1000.fasta  -o
maker2_rnd2_maker -l /usr/bin/busco/metazoa_odb9/ \
  -m genome -c 8 --long -sp fly -z --augustus_parameters='--
progress=true'
```
We also get a glimpse of our “completeness” - here we had C91.5% [S:90.1%,D:1.4%],F:2.6%,M:5.9%,n:978
This produces a directory 'run_maker2_rnd2_maker/augustus_output/retraining_parameters
Now we rename the output files. ***This re-naming step is vital, or Augustus won’t find anything:
```
rename 's/BUSCO_maker2_rnd2_maker_2594518820/agra_round2/g' *
sed -i 's/BUSCO_maker2_rnd2_maker_2594518820/agra_round2/g'
agra_round2_parameters.cfg
sed -i 's/BUSCO_maker2_rnd2_maker_2594518820/agra_round2/g'
agra_round2_parameters.cfg.orig1
```

Now these need to be copied into the AUGUSTUS HMM directory so they can be located by Augustus. Make sure you copy to the version of Augustus called by maker specifically (check the maker_exe.ctl file).
Make a new directory within augustus/config/species.
*** The name of this directory MUST MATCH the training files. These training files were "agra_round2"

```
cp agra_round2* /home/.../augustus-3.2.2/config/species/agra_round2
```

In subsequent augustus runs, SPECIES=agra_round2

Count the number of gene models after each round of maker:

```
cd maker1.maker.output
cat maker1.all.maker.gff | awk '{ if ($3 == "gene") print $0 }' | awk
'{ sum += ($5 - $4) } END { print NR, sum / NR }'
```

And view a histogram of AED scores to mark improvement:

```
perl /home/scutopus/maker/bin/AED_cdf_generator.pl -b 0.025
maker1.all.maker.gff
```

Output: 39488 6450.59

Maker Run 2:
Used the same parameters as run 1, but incorporated the training files for both SNAP and Augustus as listed above.

Running trained Augustus on its own:

```
augustus --gff3=on --outfile=augustus.gff3 --species=agra_round2
scaffolds.fasta
```

PASA Run 1:

```
/share/apps/pasa/PASApipeline-v2.3.3/Launch_PASA_pipeline.pl \
    -c alignassembly.config -C -R -g ../4addedscaffolds.fasta \
    -t Acanthopleura_granulata_KK701_composite_cd-hit.fasta.clean -T -
u Acanthopleura_granulata_KK701_composite_cd-hit.fasta \
    --ALIGNERS blat,gmap --CPU 1
### Changed CPU to 1 because of known issues using SQLite and
multithreading
```

Using EVM to move from .gff3 to CDS:

```
use evm
srun --mpi=pmi2 -n 1  *.gff3 ../4addedscaffolds.fasta CDS > CDS.fasta
```

Combining features from multiple annotations:

```
bedtools merge -i *.gff3
```

Transdecoder:

```
#!/bin/bash
for FILENAME in *.fasta
do
TransDecoder.LongOrfs -t $FILENAME
done

for FILENAME in ./*/longest_orfs.pep
do
DIR=`echo $FILENAME | cut -d "/" -f 1-2`
echo $FILENAME
echo $DIR
blastp -query $FILENAME -db
/home/wirenia/blast_dbs/uniprot_sprot_21_Sept_2016/uniprot_sprot.fasta
-max_target_seqs 1 -outfmt 6 -evalue 1e-5 -num_threads 30 -out
$DIR/longest_orfs.pep.blast_results
done

for FILENAME in ./*.transdecoder_dir/longest_orfs.pep
do
hmmscan --cpu 30 --domtblout $FILENAME.domtblout
/home/wirenia/blast_dbs/Pfam27/Pfam-A.hmm $FILENAME
done

for FILENAME in *.fa
do
TransDecoder.Predict --cpu 30 -t $FILENAME --retain_long_orfs --
single_best_orf --retain_pfam_hits
./$FILENAME.transdecoder_dir/longest_orfs.pep.domtblout --
retain_blastp_hits
./$FILENAME.transdecoder_dir/longest_orfs.pep.blast_results
done
```

Genomic Comparisons:

RepeatModeler:
Run on all downloaded mollusc genomes for comparisons:

```
use repeatmodeler-1.0.8

BuildDatabase -name *.seqfile -engine ncbi *-scaffolds.fas
RepeatModeler -database *.seqfile >& *.seqfile.out
```

REAPR:
Using REAPR on trimmed PE data to determine genome completeness:

```
reapr smaltmap -n 2 $GENOME $PE_1 $PE2 $GENOME.mapped.bam
reapr perfectmap reapr_renamed_4added.fa AGRA_1.fastq AGRA_2.fastq 150
perfect
```

GenomeScope:
Obtaining reads.histo files from jellyfish for other molluscan genomes for GenomeScope:

```
use trinity
me=`whoami`
fastq-dump --defline-seq '@$sn[_$rn]/$ri' --split-files SRR*******
rm -rf /home/$me/ncbi/
srun jellyfish count -C -m 21 -s 100M -t 12 *.fq -o reads.jf
srun jellyfish histo -t 12 reads.jf > reads.histo
```

Salmon:
Salmon mapping transcriptome(s) back to genome for quantification files to generate expression data:

```
use salmon
#index genome-based transcripts
salmon index -t Acanthopleura_granulata-transcripts.fas -i
transcripts_index -k 31
#Quantify transcriptomes
salmon quant -i transcripts_index -l A -1 KK701-*R_1.fastq.gz -2
KK701-*R_2.fastq.gz --validateMappings -o KK701.*.transcripts_quant
```

CDHit and CDHit-EST:

```
cdhit -i Combined.fasta -o cdhitCombined.fas -n 4 -c 0.8 -T 10
cdhit-est -i $file -o $file.0.8.cdhit -c 0.8 -T 10
```

SIRE:
Uploaded each taxon's clustered file to SIRE2.0 WebServer

IRE 5' or 3':

```
taxa=

#First make a new .gff3 file with only the high quality IRES
grep "quality=High" *.gff3 > $taxa.hqIREs.gff

paste <(cut -f 1,4 *hqIREs.gff) > $taxa.hqIRE.start.txt

#Now produce lengths for each transcript:
awk '$0 ~ ">" { print c ; c=0 ; printf substr($0,2,100) "\t" } $0 !~
">" { c += length($0) } END {print c }' *transcripts.fas >
temp.transcriptlengths.txt
#it accidentally added a blank line at the beginning. This is faster
than fixing it:
awk NF temp.transcriptlengths.txt > $taxa.alltranscriptlengths.txt
rm temp.transcriptlengths.txt

#Grep out the transcripts that contain an IRE:
awk '{ print $1 }' $taxa.hqIRE.start.txt > IREtranscripts.txt

#looping through to permit duplicates:
while read p; do
  grep $p $taxa.alltranscriptlengths.txt >>
$taxa.IREtranscriptlengths.txt
done <IREtranscripts.txt

awk '{ print $1, ($2/2) }' $taxa.IREtranscriptlengths.txt >
$taxa.MIDDLES.txt

#Now make a new table:
paste <(cut -f 1,2 $taxa.MIDDLES.txt) <(cut -f 2
$taxa.hqIRE.start.txt) > Name.Middle.IRE.txt

awk '$2<$3 {print $1,$2,$3,"3prime"}' Name.Middle.IRE.txt > 3or5.txt

awk '$2>$3 {print $1,$2,$3,"5prime"}' Name.Middle.IRE.txt >> 3or5.txt

echo -e $taxa.HQIREs' \t '$taxa.3prime' \t '$taxa.5prime >
$taxa.totalIREs.txt
```

Ortholog Searching:

```
orthofinder -t 20 -I 2.1 -M msa -T fasttree -f ./pep -o
./OrthoFinder_results
```

OrthoFinder CleanUp:

```
#Delete sequences shorter than $MIN_SEQUENCE_LENGTH
echo "Deleting sequences shorter than $MIN_SEQUENCE_LENGTH AAs..."
for FILENAME in *.fa
do
grep -B 1 "[^>].\{$MIN_SEQUENCE_LENGTH,\}" $FILENAME > $FILENAME.out
sed -i 's/--//g' $FILENAME.out
sed -i '/^$/d' $FILENAME.out
rm -rf $FILENAME
mv $FILENAME.out $FILENAME
done
echo Done

#If fewer than $MIN_TAXA different species are represented in the
file, move that file to a "rejected_few_taxa" directory.
echo "Removing groups with fewer than $MIN_TAXA taxa..."
mkdir -p rejected_few_taxa_1
for FILENAME in *.fa
do
awk -F"|" '/^>/{ taxon[$1]++ } END{for(o in taxon){print o,taxon[o]}}'
$FILENAME > $FILENAME\.taxon_count #Creates temporary file with taxon
abbreviation and number of sequences for that taxon in $FILENAME
taxon_count=`grep -v 0 $FILENAME\.taxon_count | wc -l` #Counts the
number of lines with an integer >0 (= the number of taxa with at least
1 sequence)
if [ "$taxon_count" -lt "$MIN_TAXA" ] ; then
echo $FILENAME
mv $FILENAME ./rejected_few_taxa_1/
fi
done
rm -rf *[0-9].fa.taxon_count
echo Done
echo


#Remove redundant sequences using uniqHaplo
(http://doi.org/10.5281/zenodo.166024)
mkdir preUniqHaplo
cp *.fa preUniqHaplo
echo "Removing redundant sequences using uniqHaplo..."
```

```
ls *[0-9].fa | parallel -j $CORES 'perl /usr/bin/uniqHaplo.pl -a {} >
{}.uniq'
rm -rf *.fa
rename 's/.fa.uniq/.fa/g' *.fa.uniq
echo Done
echo

#Align the remaining sequences using Mafft.
echo "Aligning sequences using Mafft (auto)..."
mkdir backup_alignments
ls *[0-9].fa | parallel -j $CORES 'mafft --auto --localpair --
maxiterate 1000 {} > {}.aln'
rm -rf *[0-9].fa
rename 's/.fa.aln/.fa/g' *.fa.aln
cp *.fa ./backup_alignments/
echo Done
echo

#Remove newlines.
echo "Removing linebreaks in sequences..."
for FILENAME in *.fa
do
sed -i ':a; $!N; /^>/!s/\n\([^>]\)/\1/; ta; P; D' $FILENAME
done
echo Done
echo

#Clean alignments with HmmCleaner
echo "Removing misaligned sequence regions with HmmCleaner..."
mkdir HmmCleaner_files
ls *.fa | parallel -j $CORES 'HmmCleaner.pl {} --specificity'
mv *.fa ./HmmCleaner_files
mv *.log ./HmmCleaner_files
mv *.score ./HmmCleaner_files
rename 's/_hmm.fasta/.fa/g' *_hmm.fasta
echo Done
echo


#Trim alignments with BMGE
echo "Trimming ambiguously aligned columns in the alignment with
BMGE..."
mkdir backup_pre-BMGE
ls *.fa | parallel -j $CORES 'java -jar /usr/bin/BMGE-1.12/BMGE.jar -i
{} -t AA -of {}.BMGE'
```

```
mv *.fa ./backup_pre-BMGE
cp *.BMGE ./backup_pre-BMGE
rename 's/.BMGE//g' *.BMGE
echo Done
echo

#Remove newlines.
echo "Removing linebreaks in sequences..."
for FILENAME in *.fa
do
sed -i ':a; $!N; /^>/!s/\n\([^>]\)/\1/; ta; P; D' $FILENAME
done
echo Done
echo

#Remove any sequences that don't overlap with all other sequences by
at least 20 amino acids.
for FILENAME in *.fa
do
java -cp /usr/bin AlignmentCompare $FILENAME
done
echo Done
echo
rm -rf myTempFile.txt

#If fewer than $MIN_TAXA different species are represented in the
file, move that file to the "rejected_few_taxa" directory.
echo "Removing groups with fewer than $MIN_TAXA taxa..."
mkdir -p rejected_few_taxa_2
for FILENAME in *.fa
do
awk -F"|" '/^>/{ taxon[$1]++ } END{for(o in taxon){print o,taxon[o]}}'
$FILENAME > $FILENAME\.taxon_count #Creates temporary file with taxon
abbreviation and number of sequences for that taxon in $FILENAME
taxon_count=`grep -v 0 $FILENAME\.taxon_count | wc -l` #Counts the
number of lines with an integer >0 (= the number of taxa with at least
1 sequence)
if [ "$taxon_count" -lt "$MIN_TAXA" ] ; then
echo $FILENAME
mv $FILENAME ./rejected_few_taxa_2/
fi
done
rm -rf *.fa.taxon_count
echo Done
echo
```

```
#Makes a tree for each OG with FastTree
echo "Making a tree for each OG using FastTreeMP..."
for FILENAME in *.fa
do
FastTreeMP -slow -gamma $FILENAME > $FILENAME.tre
done
rename 's/.fa.tre/.tre/g' *.fa.tre
echo Done
```

PhyloPyPruner:

```
#Runs PhyloPyPruner 0.9.5
echo "Running PhyloPyPruner..."
phylopypruner --threads $CORES --dir . --min-taxa $MIN_TAXA --min-len
$MIN_SEQUENCE_LENGTH --min-support 0.75 --mask pdist --trim-lb 3 --
trim-divergent 0.75 --min-pdist 0.01 --trim-freq-paralogs 3 --prune MI
cd phylopypruner_output
FastTreeMP -slow -gamma supermatrix.fas > FastTree.tre
cd ..
```

Extracellular Localization Signals:

Uploaded the set of 31 genes from SilkSlider as a .fa file to SignalP 5.0 WebServer

Genome Comparison Plot:

```
## R Markdown

#Import "GenomeQualityNum"

d <- GenomeQualityNum[, c("Num", "BUSCO", "N50")]
#REMEMBER: for color coding, category MUST BE A NUMBER

#Better markers
pdf("GenomeQualities_3.pdf", width=8, height=8)
plot(d$BUSCO, d$N50, log="y", xlim=c(15,100), ylim=c(0.001,1000),
xlab="BUSCO score, %", ylab="log(scaffold N50), Mb",
pch=ifelse(d$Num==1,  19 ,ifelse(d$Num==2, 8, ifelse(d$Num==3, 17,
ifelse(d$Num==4, 15, 18)))), frame.plot=FALSE, col=ifelse(d$Num==1,
"darkorange3" ,ifelse(d$Num==2, "royalblue2", ifelse(d$Num==3,
"mediumpurple1", ifelse(d$Num==4, "darkolivegreen3", "grey")))))
dev.off()
```

Heatmaps of Gene Expression:

```
library(pheatmap)
library(RColorBrewer)
####################
```

```
Getting rid of all-0 rows

df[rowSums(df[, -1])>0, ]
nonzeroTPMIRE <- logTPMIRE[rowSums(logTPMIRE[, -1])>0, ]

pheatmap (nonzeroTPMIRE, cluster_rows=T, cluster_cols=F,
show_rownames=F, show_colnames=F, color =
RColorBrewer::brewer.pal(9,"YlGnBu"))
```
